# A qPCR-duplex assay for sex determination in ancient DNA

**DOI:** 10.1371/journal.pone.0269913

**Published:** 2022-06-10

**Authors:** Anna Poma, Patrizia Cesare, Antonella Bonfigli, Anna Rita Volpe, Sabrina Colafarina, Giulia Vecchiotti, Alfonso Forgione, Osvaldo Zarivi

**Affiliations:** 1 Department of Life, Health and Environmental Sciences, University of L’Aquila, L’Aquila, Italy; 2 Department of Human Studies, University of L’Aquila, L’Aquila, Italy; University of Florence, ITALY

## Abstract

Molecular biology techniques are increasingly being used in sex identification of skeletal remains when traditional anthropometric analyzes are not successful in identifying sex of remains that are incomplete, fragmented and /or of immature individuals. In the present work, we investigated the possibility of determining sex by using the qPCR-duplex method for both ancient and modern DNA samples. This method involves the co-amplification of two genes in a single reaction system and the subsequent analysis of the fusion curves; the gene sequences used for the construction of suitable primers are those of steroid sulfatase (STS) and testis specific protein Y-linked 1 (TSPY) genes which turned out to be two sensitive markers as they have a detection limit of 60 pg and 20 pg respectively on modern DNA. The validity of the method was verified on modern DNA in which gender was identified in all the samples with 100% accuracy; thus, allowing for the same results as the classic method with amelogenin, but in a faster and more immediate way, as it allows for sex determination solely by analyzing the denaturation curves without having to perform an electrophoretic run. The proposed molecular technique proves to be sensitive and precise even on degraded DNA, in fact on 9 archaeological finds dating from the VII-XII century in which sex had been identified through anthropometric analysis, it confirmed the sex of 8 out of 9 finds correctly.

## Introduction

Sex determination of archaeological human remains is of fundamental importance for anthropological studies of ancient societies, cultures and genealogical histories. So far, determining sex of human remains has been based on the sexual dimorphism present in most human bones [1–3].

However, anthropometric studies provide good results only when the skeletons are complete, in good condition and of adult individuals [4,5].

The advent of molecular genetics has allowed for the development of more sensitive methods to determine sex. Polymerase chain reaction (PCR) is a fast and inexpensive procedure that allows for the amplification of single target DNA molecules in analytical quantities. With the development of the polymerase chain reaction (PCR), DNA analysis to determine sex can be performed using small amounts of DNA in the forensic field and on various biological artifacts, including bones and archaeological remains [6–8].

The genetic difference between male and female is determined by the presence of a Y chromosome in males, so the detection of specific sequences on this chromosome allows us to distinguish between male and female individuals. DNA-based gender identification can be performed using various sex-specific markers such as AMEL, SRY; TSPY; DXYS156; and DYZ1 which are located on the Y chromosome [9,10].

Although there are numerous markers for determining sex, the most used is the amelogenin gene, both on modern biological samples and for anthropological studies on ancient DNA (aDNA); the amplification of the amelogenin gene produces one amplicon on female DNA and two amplicons on male DNA [5,11–20], however there are some critical issues with this method due to the fact that the amelogenin gene can be deleted on the Y chromosome [21].

Furthermore, in archaeological finds, exogenous contamination and fragmentation of extracted DNA can be critical factors in determining sex [22,23]. To overcome fragmentation of ancient DNA, researchers have generated small amplicons in quantitative real-time polymerase chain reaction (qPCR). Existing literature on the determination of sex/gender in fragmented DNA includes studies that involve a qPCR-simplex followed by the analysis of the fusion curve [24,25]; in this study we have developed a method of qPCR-multiplex followed by the analysis of the fusion curve that is robust, fast and sensitive.

The qPCR-duplex approach involves the co-amplification of two genes in a single reaction system, the identification of a single peak or two distinct peaks in the thermal denaturation curves allows us to define whether the DNA template is female or male [26,27].

This method does not require any electrophoretic processing or separation after qPCR thus a considerable numbers of samples can be analysed at the same time with high-level performance.

To identify human sex in this work we used a qPCR-duplex amplification of the TSPY and STS genes, which respectively encode the Testis-specific Y-encoded protein 1 and Steroid sulfatase.

TSPY belongs to a heterogeneous family of genes specific for the Y chromosome, which in humans maps in the Yp11.2 region even if there are some copies on the long arm Yq [28].

A large TSPY matrix was found in the proximal part of Yp, while a smaller repeating matrix was found in the most distal part [29]; the number of copies in the human genome varies on an individual basis but about 35 copies have been estimated, thus making this gene a more sensitive marker than others for sex identification in archaeological finds.

This high copy-number characteristic makes it very sensitive and therefore a functional marker when dealing with very small quantities of DNA or with significantly degraded samples, or in the case of mixtures of male and female DNA.

A study on the sensitivity of the TSPY marker confirmed that this gene can give results starting from 4 pg of DNA, while the SRY marker only starting from 80 pg [30].

All the characteristics listed above make this gene a hypothetically successful marker for sex identification of forensic samples and of highly degraded archaeological finds.

The STS gene is located on the short arm of the X chromosome, a pseudogene is present on Yq11.211 and is designated STSP1 (Steroid Sulfatase Microsomial Pseudogene 1) [31]. The sequence homology between STS and STSP1 is 84.5%, however, STSP1 does not encode a functional protein [32] The STSP1 pseudogene can potentially be used for sex typing and can be successfully amplified in male specimens.

By amplifying the sequences, in the same reaction system, of both the TSPY gene and the STS gene, the qPCR-duplex allowed us to immediately identify the sex through the analysis of the thermal denaturation curves of the amplicons we had produced.

This work also presents preliminary data aimed at evaluating whether the proposed method can be used for determining sex on ancient DNA samples, in fact the short length of the amplicons produced in qPCR on the TSPY (67 bp; 119 bp) and STS (89 bp; 95 bp; 116 bp; 154 bp; 158 bp) and the high copy / genome number of the TSPY gene [33], facilitated the determination of sex of degraded and low concentration samples.

The concentration of DNA in human archaeological remains was estimated, sensitively by means of a standard curve, constructed by amplifying the consensus sequence Alu in qPCR in a control DNA; Alu is a highly repetitive sequence and specific to humans [34–36].

## Materials and methods

### Sampling and ethics statement

Bone samples used were collected by Profs. Fabio Redi, and Alfonso Forgione, responsible for the archaeological excavations, and are stored in the Archaeology Laboratory of the Department of Human Studies, University of L’Aquila, L’Aquila, Italy. Under Italian law, and in compliance to current regulations, no permits were required for the study described.

Peripheral blood samples were collected from the authors of this article and from volunteers, prior written informed consent. The subjects authorized the use of the samples solely for the purpose of positive controls of sex no other scientific research objectives involved. All blood samples have not been and will not be used for any other purpose. Personal identification information is not associated to the samples.

### DNA extraction from blood samples

Peripheral blood samples (50–100 μl) were collected from Italian volunteers: 10 males and 8 females; prior informed consent that the samples be used only as a gender control with no other scientific research intents. DNA was extracted using the GeneAll® ExgeneTM DNA micro kit following the manufacturer’s instructions, eluted in 10mM Tris-HCl (pH 8.0), 1mM EDTA and checked with agarose gel electrophoresis.

The DNA concentration in the extracts was determined using Thermo Fisher’s Qubit ™ 4 fluorometer. Purity in all samples was evaluated based on absorbance ratios A260/A280 e A260/230 using a Nanodrop 2000 spectrophotometer (Thermo Scientific, Waltham, WA).

A mixture of DNA extracted from 4 males and 4 females (control DNA) was used to construct the standard ALU_50/127 curve.

### Recovery of skeletal remains for the extraction of aDNA

The aDNA samples were obtained from remains of human bones dating back to the 8th, 9th and 12th century AD, found in the Cathedral of Santa Maria in Civitate in Amiternum L’Aquila, Italy during the 2016/17 archaeological expeditions [37–39]. Recovery of the remains was carried out by researchers from the Archeology Laboratory and in the presence of a molecular biologist from the Laboratory of Genetics and Mutagenesis respectively employed in the Departments of “Human Studies” and “Life Health and Environment Sciences” of the University of L’Aquila.

The molecular biologist developed the protocol to ensure that all necessary precautions were taken during the excavation and recovery of the remains in order to avoid any type of contamination of the samples with modern DNA [40,41]; samples for aDNA extraction and biomolecular analyzes were taken from the bone remains prior to any anthropological analysis.

Following light brushing with a dry brush, the samples were packaged separately in plastic bags and transported in airtight containers to the genetics and mutagenesis laboratory where they were stored at -20° C until use. All the material used was pre-treated with 10% bleach.

Table 1 shows: the burial period, the age of the individuals at the time of death and gender determined with standard methods applied in physical anthropology. The remains come from five intact graves (S.46, S.43, S.50, S.48 and S.49) and four altered but well-preserved graves (S.44, S.42, S.45 and S. 47).

**Table 1:** Skeletal remains analyzed.

Samples	Bone	Age (years)	Sex	Period
S.42	Right Femur	45–50 (Adult)	Male	9th-10th centuries
S.43	Right Femur	50–60 (Adult)	Male	12th centuries
S.44	Right Femur	45–50 (Adult)	Female	9th-10th centuries
S.45	Right Femur	45–50 (Adult)	Male	9th-10th centuries
S.46	Left Femur	16–18 (Teenager)	Male	9th-10th centuries
S.47	Left Femur	55–60 (Adult)	Female	9th-10th centuries
S.48	Left Femur	45–50 (Adult)	Male	8th centuries
S.49	Right Femur	60–50 (Adult)	Female	8th centuries
S.50	Right Femur	45–50 (Adult)	Female	12th centuries

### DNA extraction from bone findings

The extraction of ancient DNA was carried out following the guidelines for the analysis of human skeletal remains to prevent environmental contamination. The experiments were conducted in two separate laboratories equipped with dedicated equipment: in the pre-PCR laboratory, all the experimental phases preceding the amplification of ancient DNA are performed; in the post-PCR laboratory all experimental phases with DNA already amplified and no longer at risk of contamination are performed [40–44]. In the pre-PCR laboratory, all experiments were carried out by two researchers equipped with special devices; all surfaces were carefully treated with 10% bleach and irradiated with UV rays at 254nm for one night before use. Before each experiment all equipment was sterilized with 6% bleach and, if necessary, with ethanol and then irradiated for 4 hours with UV rays at 254 nm [45–47]. Bone powder was obtained by milling a small area of the bone findings with a mini DEXTER, at a speed of 800 rpm, this area was previously washed with 6% bleach in sterile water and exposed to ultraviolet rays for two cycles of 20 min in a Hoefer Ultraviolet UVC 500 at 254 nm. With the first milling about 2 mm of bone are removed and discarded; after subsequent milling the pulverized bone samples (about 400 mg) were recovered in a sterile Falcon tube and then stored at -20°C until their use. DNA was extracted using PrepFiler™ BTA Forensic DNA Extraction Kit developed for the extraction of DNA from calcified tissues (bone and tooth) (Applied Biosystems), following the manufacturer’s instructions. Three DNA extractions were performed for each sample and the concentration of each sample was calculated using the standard Alu curves [34,35].

### Primers: Design and expected amplicons

Two target gene sequences have been identified to establish the male or female origin of the samples examined, the TSPY gene and the STS gene.

The sequence of the amelogenin gene was also taken into consideration for the determination of the sex. The sequence corresponding to the Alu element was used to quantify ancient DNA.

The TSPY gene is present with about 35 copies in the human genome, it therefore constitutes a sensitive and elective marker when DNA concentrations are low or degraded. The STS gene is present on the X chromosome, while on the Y chromosome a pseudogene called STSP1 is present which shows sequence homology with the STS gene of about 84.5%. STSP1 does not encode a functional protein but it can be used for sex determination, in fact the amplification of regions with deletions allows for distinguishing the Y chromosome from the X.

The study of the sequences of these genes allowed us to identify nucleotide regions where to build primers (forward primer and reverse primer) capable of producing amplicons of different sizes with distinct melting temperatures.

Amelogenin is a gene that encodes a protein forming tooth enamel. The gene is present on both the Y and X chromosome, but on the latter it has a deletion of 6 bp, therefore the amplification product with the primers chosen on the X and Y chromosome are of 106 bp and 112 bp, respectively.[12].

The consensus sequence Alu used for the design of the primers has a size of ~ 280 bp. The amplification of this sequence in PCR allows us to detect quantities of DNA from 128 ng/μl to 0.5 pg/μl [34,48] and therefore to quantize the DNA in low concentration samples, and also to detect contamination of the samples with human DNA [49]. The ***Alu_127*** primers were taken from existing literature [12,35]. The primers on the STS, STSP1, TSPY and ALU_50 genes were designed with the Primer Express 3.0 software (Applied Biosystems, USA), using human sequences with the following access numbers: TSPY NC_000024.9, NG_027958.1, NT_011878.9; STS NC_000023.10, NG_021472.1, NT_167197.1; STSP1 ENSG00000227166.

The predicted amplification product and the specificity of primers were checked using UCSC Genome Browser online software (http://genome.ucsc.edu/cgi-bin/hgPcr). In order to obtain broader divergence between melting peaks, amplicons melting temperature (Tm) was predicted using Oligo Calculator software (http://www.basic.northwestern.edu/biotools/oligocalc.html). The melting temperatures (Tm) and the thermal denaturation curves of the amplicons were predicted using the uAnalyze V2 software (https://dna-utah.org/ua/uanalyze.html).

Validation studies on male and female DNA extracted from blood samples were performed for all selected primers; the results allowed us to determine: the amplification efficiency; the coefficient of determination R2; the sensitivity of amplification of male and female DNA in the system; the number of amplicons produced. Furthermore, we analysed the thermal denaturation curves and, through electrophoresis, the amplification products (S1 File). Table 2 shows: the primers used on the basis of validation studies, the Tm of the individual primers, the size of the expected amplicons and amplification efficiency in both sexes. The ***AMEL106/112*** primers, for the amplification of the amelogenin gene were taken from existing literature [12,35].

**Table 2:** List of primers used.

PRIMERS	PRIMER	PRIMER SEQUENCE 5’-3’	Tm	PRODUCT (bp)	Efficiency	
					♂	♀
***ALU260***	FORW_ALU260 REV_ALU260	CTCACGCCTGTAATCCCAGC GAGTCTCGCTCTGTCGCC	59.2°C 58.7°C	260	98%	98%
***ALU127*** *	FORW_ALU127 REV_ALU127	GACCATCCCGGCTAAAACG CGGGTTCACGCCATTCTC	58.8°C 58.2°C	127	98%	98%
***ALU50***	F_ALU_50 R_ALU_50	GATCACGAGGTCAGGAGGTC GGGTTTCACCGTTTTAGCCG	61,4°C 59,4°C	50	101%	102%
***TSPY67***	FORW_TSPY67/119 REV_TSPY67	GCTTCTCATTCCACTCCAATTGA GGTGTCTGCGGCGATAGG	59.8°C 60.5°C	67♂ - ♀	99%	
***TSPY119***	FORW_TSPY67/119 REV_TSPY119	GCTTCTCATTCCACTCCAATTGA CCTGCGAAGTTGTGGTCAGA	59.8°C 59.4°C	119♂ - ♀	101%	
***STS89***	FORW_STS95_89 REV_STS89	CATTCATAGAGAAAGGCTAAAGGTATCA CCTTTGCTGGCTTATATTTTTTATGC	60.7°C 58.5°C	89 ♂ 89 ♀	99%	103%
***STS95***	FORW_STS95_89 REV_STS95	CATTCATAGAGAAAGGCTAAAGGTATCA ACATACCCTTTGCTGGCTTATATTTT	60.7°C 58.5°C	95 ♂ 95 ♀	98%	98%
***STS158Y***	FOR_STS158Y REV_STS158Y	GCACCAGGGTGGCTTATCTT TATGCTCCAGGGAAAACGCA	59.4°C 57.3°C	158 ♂ - ♀	101%	
***STS154/116***	FOR_STS 154/116 REV_STS 154/116	TCACAGCCTCTTCGGGAG ACTTTGTCCAAGTCCTCCCT	58.2°C 57.3°C	154–116 ♂ 154 ♀	103%	98%
***STS120***	FORW_1STS120 REV_1STS120	TGGTTGTGGGATTCCCTTTG GCTTCACTCTTCTCCGATCCA	57.3°C 59.8°C	120 ♂ 120 ♀	98%	99%
***AMEL106/112*** *	For_AMEL106/112 Rev_AMEL106/112	CCCTGGGCTCTGTAAAGAA ATCAGAGCTTAAACTGGGAAGCTG	61,6°C 62,2°C	106–112 ♂ 106 ♀	99%	98%

* primers taken from literature.

The primers were chosen in order to distinguish male from female DNA (S1 Fig). The FORW_STS_95 89 primer was designed on the STS gene straddling a deletion on the X chromosome and unable to pair with the STSP1 pseudogene, therefore the ***STS95*** and ***STS89*** primers can only amplify the X allele (S1A Fig; Table 2).

The FOR_STS158Y primer was designed on the STSP1 pseudogene in a portion of the deleted sequence in the STS gene, therefore the ***STS158Y*** primers can only amplify the Y allele (S1B Fig; Table 2).

The ***STS154 /116*** primers were constructed on the STS gene sequence in a portion where the STSP1 pseudogene has two deletions. The amplicons produced will be of 154 bp on the X allele and of 116 bp on the Y allele, hence an electrophoretic running will highlight two bands on the male DNA and one band on the female DNA (S1C Fig; Table 2).

The REV1STS120 primer was designed on the STS gene in a region where there is a deletion on the STSP1 pseudogene and therefore the ***STS120*** primers can only amplify the X allele (S1D Fig; Table 2).

For the TSPY gene, two primers ***TSPY119*** and ***TSPY67*** were constructed producing respectively an amplicon of 119 and 67 bp on the Y allele (S1E Fig; Table 2).

### Silico analysis of melting curves

The in-silico analysis of the melting curves of the expected amplicons is a simple and powerful method that is used in many applications of molecular biology [50]. UAnalyze SM software (https://dna-utah.org/ua/uanalyze.html) generates melting curves by setting: the temperature range; concentrations of monovalent cations and Mg^++^.

The sequences entered on the platform are analyzed by the software that performs the thermodynamic calculations generating the melting curves and the theoretical Tm. Subsequently, the primers selected were experimentally tested to define optimal concentrations and to verify the number and size (bp) of amplicons produced in both qPCR and qPCR-duplex reactions. The coefficient of efficiency in qPCR was then determined for each primers by amplifying the male and female DNA extracted from the blood samples. The screening carried out allowed for a rigorous evaluation of the primers thus we were able to eliminate from the study the primers that did not respect the parameters we had set.

### Quantitative real-time PCR and PCR

qPCR was performed with a PCI ABI 7300 (Applied Biosystem) instrument in 96-well plates, using SYBR Green to detect DNA synthesis. The reaction mix (20 μl total volume) contained: 10 μl of PowerUp "SYBR" Green Master Mix (2x) (Applied Biosystems), 2 μl mix of specific forward and reverse primers (5 μM), DNA at various concentrations and ddH_2_O variable to up 20 μl.

The thermocycler program consisted of an UDG activation at 50°C for 2 minutes, an initial heat activation step at 95 °C for 2 min, followed by 40 cycles at 95 °C for 15 sec and 60 °C for 1 min, and an analysis of the melting curve with a temperature ramp from 60°C to 95°C.

At the end of the process the amplification and melting curves (60–95°C) were analyzed with the ABI 7300 system software. All the analyzed samples showed: specific melting peaks; PCR efficiency greater than 90%; satisfactory quality standard curves (R^2^ ~ 1); high precision of quantification values. Negative controls are always included in each amplification (no DNA in the system). To determine the relative level of inhibitors present in the extracts, shifts in the Ct value for the internal PCR control (IPC) in the real-time PCR assay were checked. All samples were amplified in triplicate. PCR was conducted with the Hybaid PCR Express ThermoCycler instrument using the KAPA2G Fast HotStart ReadyMix 2X kit (Kbiosystems).

### qPCR-duplex

The qPCR-duplex reactions were performed in a final volume of 20 μl containing: 10 μl of PowerUp "SYBR" Green Master Mix (2x) (Applied Biosystems), 2 μl of a specific pair of primers at the concentrations shown in Table 3, DNA at various concentrations and variable ddH2O up to 20 μl. The thermal cycler program consisted of a UDG activation at 50° C for 2 minutes, an initial heat activation step at 95° C for 2 min, followed by 40 cycles at 95° C for 15 sec and 60° C for 1 min and an analysis of the melting curve with temperature ramp from 60° C to 95° C. At the end of the process the amplification and melting curves (60–95° C) were analyzed with the ABI 7300 system software.

**Table 3:** Pairs of primers used in qPCR-duplex. Optimal concentrations and expected amplicons in both sexes are reported for the various primers.

Pairs of Primers	Concentrations of Primers	Sex	Amplicons
***STS158*** ***STS89***	300 mM 400 mM	♀	89 bp
		♂	158 bp /89 bp
***STS89*** ***TSPY119***	600 mM 200 mM	♀	119 bp
		♂	89 bp /119 bp
***STS95*** ***TSPY67***	400 mM 200 mM	♀	95 bp
		♂	95 bp /67 bp

### Construction of standard Alu curves and quantification of DNA in ancient bone extracts

Quantification of human aDNA in archaeological samples is one of the most important factors behind the efficiency and success of PCR-based genotyping. Alu elements are ideal sequences for detecting small amounts of DNA due to their species specificity, small size and extraordinarily high copy number [35].

The standard curve for quantifying DNA extracted from ancient bones was constructed in qPCR by amplifying the Alu sequence in control DNA. The qPCR was performed on six 1:10 serial dilutions from 29 ng / μl to 0.29 pg / μl of the sample by amplifying short Alu consensus sequences with the primers ***ALU127*** (127bp) and ***ALU50*** (50 bp). qPCR reactions consisted of: 10 μl of PowerUP SYBR Green Master Mix, 2 μl Alu primers 500 nM for each primer, 1 μl of DNA extract and ddH_2_O to 20 μl.

The slope of the standard curve ALU_50 is (m = −3.332) and (R^2^ = 0.999). The slope of the standard curve ALU_127 is (m = −3.325) and (R^2^ = 0.999) (S2 Fig).

The quantity of DNA extracted from the bone remains was calculated using the standard curves; each sample was analyzed in triplicate (S1 Table).

### Reproducibility, sensitivity and accuracy of the tests

Reproducibility was tested on three biological replicates and three technical replicates were performed for each sample.

The sensitivity of qPCR was tested with a series of genomic DNA dilutions with concentrations ranging from 10 ng to 10 pg. The following statistical parameters of the threshold cycle value (Ct) were calculated: mean, standard deviation (SD).

To test the accuracy of the methods proposed in this study, DNA was extracted from 18 peripheral blood samples (50–100μl) from Italian individuals, 10 males and 8 females, and qPCR and qPCR-duplex were performed. The accuracy of the test was calculated on the ability to correctly identify the sex on the total number of the tests performed.

### Agarose gel electrophoresis

10 μl of the PCR and qPCR reactions were loaded onto a 2.2% or 2.9% low melting point agarose gel in 0.5X TAE-buffer and separated by 100 Volt electrophoresis. 0,8 μg of DNA ladder (SHARPMASS ™ 50- Ready to load DNA ladder, Euroclone S.p.A.) is used as a marker of PM. The mw of each band was estimated by comparing it to a co-migrating known size DNA ladder 100 bp (SMO321, Fermantas). The bands in the gels were revealed with 0.5 μg / ml ethidium bromide. Color gels were acquired with the Gel Doc XR+Gel Documentation System (Bio-Rad) automatic documentation system and analyzed to determine the size of the produced amplicons.

## Results and discussion

### Sex determination in modern and ancient DNA

All selected primers (***TSPY67*, *TSPY119*, *STS89*, *STS95*, *STS158Y*** and ***STS154/116***), constructed as reported in the “Materials and Methods” section of this study, were used to amplify the DNA extracted from the blood samples in qPCR and PCR.

**Fig 1:**
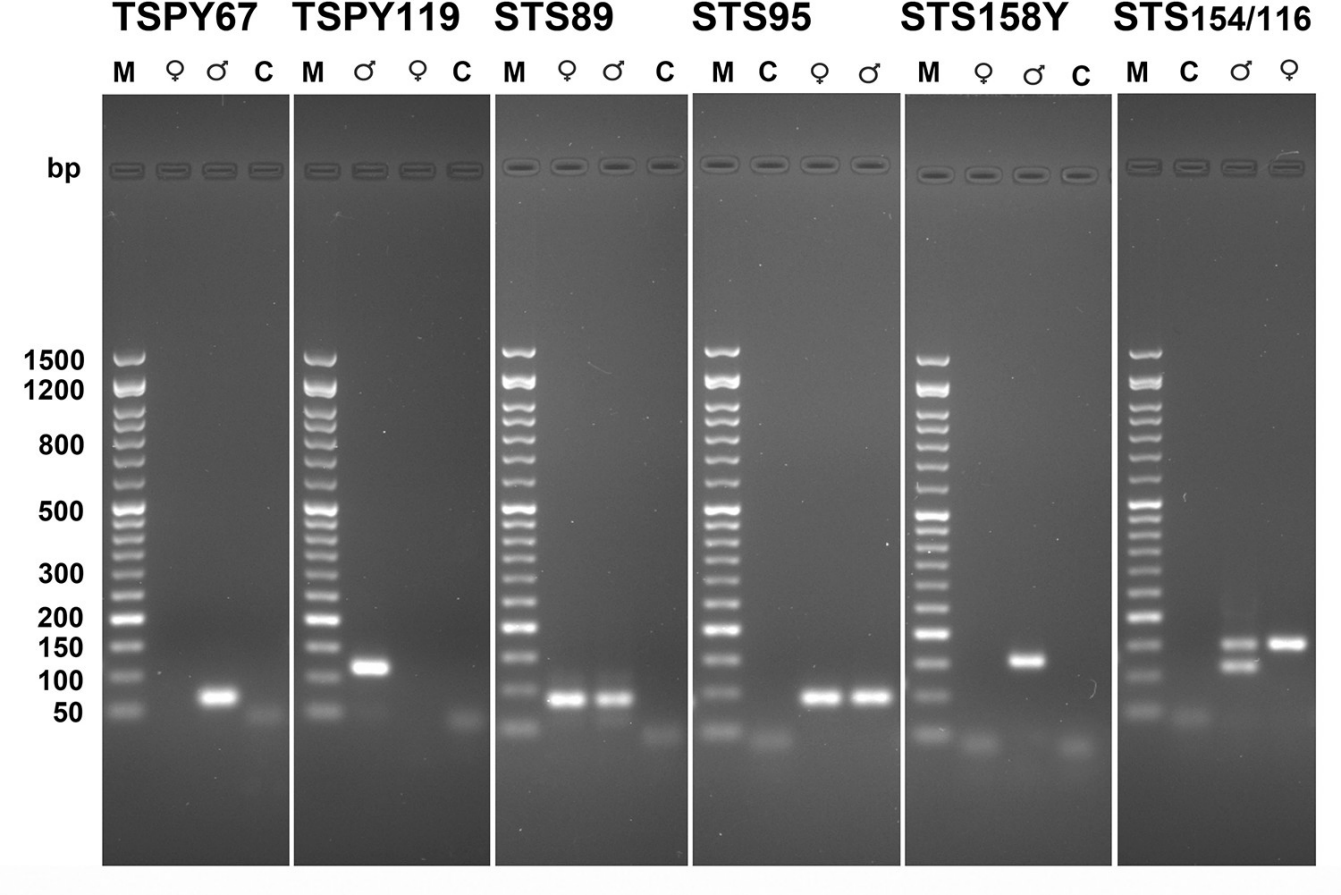
Agarose gel electrophoresis of amplicons on modern DNA. Electrophoretic analysis of amplicons generated in qPCR using the primers ***TSPY67*, *TSPY119*, *STS89*, *STS95*, *STS158Y*, *STS154/116***, with male (♂) and female (♀) human DNA. M) SharpMass ™ 50 molecular weight marker—Ready-to-load DNA Ladder (Euroclone S.p.A). C) control without template DNA. Agarose gels are 2.2% and stained with ethidium bromide as reported in Materials and Methods.

Fig 1 shows the electrophoretic runs of amplicons produced in qPCR on male and female DNA with all primers; confirming the size of the amplicons and the absence of non-specific amplification products.

The S1 File shows the amplification plots, the Ct values, the melting curves and the Tm of the produced amplicons; the thermal denaturation curves confirm the absence of non-specific amplification products.

The primers selected for the TSPY gene (***TSPY67*, *TSPY119***) amplify only the Y allele while no amplification is present on the female DNA (Fig 1 and S1 File). Fig 1 and S1 File shows that the primers selected for the STS gene (***STS89*, *STS95***) amplify only the X allele, and therefore the DNA of both sexes.

With the ***STS158Y*** primers, as expected, no amplification is observed on the female DNA due to the presence of a deletion on the STS gene of the X chromosome, while an amplicon of 158 bp is produced on the male DNA (Fig 1 and S1 File).

The results obtained with the primers ***TSPY67*, *TSPY119*, *STS89*, *STS95*** and ***STS158Y*** are shown to be able to discriminate the X and Y allele, but not to clearly distinguish the sex.

Fig 1 and S1 File shows the results obtained from the amplification of modern DNA with the ***STS154/116*** primers, the amplification plots and melting curves show amplicons on both male and female DNA (S1 File); after the electrophoretic run, two amplicons of 154 bp and 116 bp in males and a single amplicon of 154 bp in females are identified (Fig 1); the same results were obtained with classical PCR.

**Fig 2:**
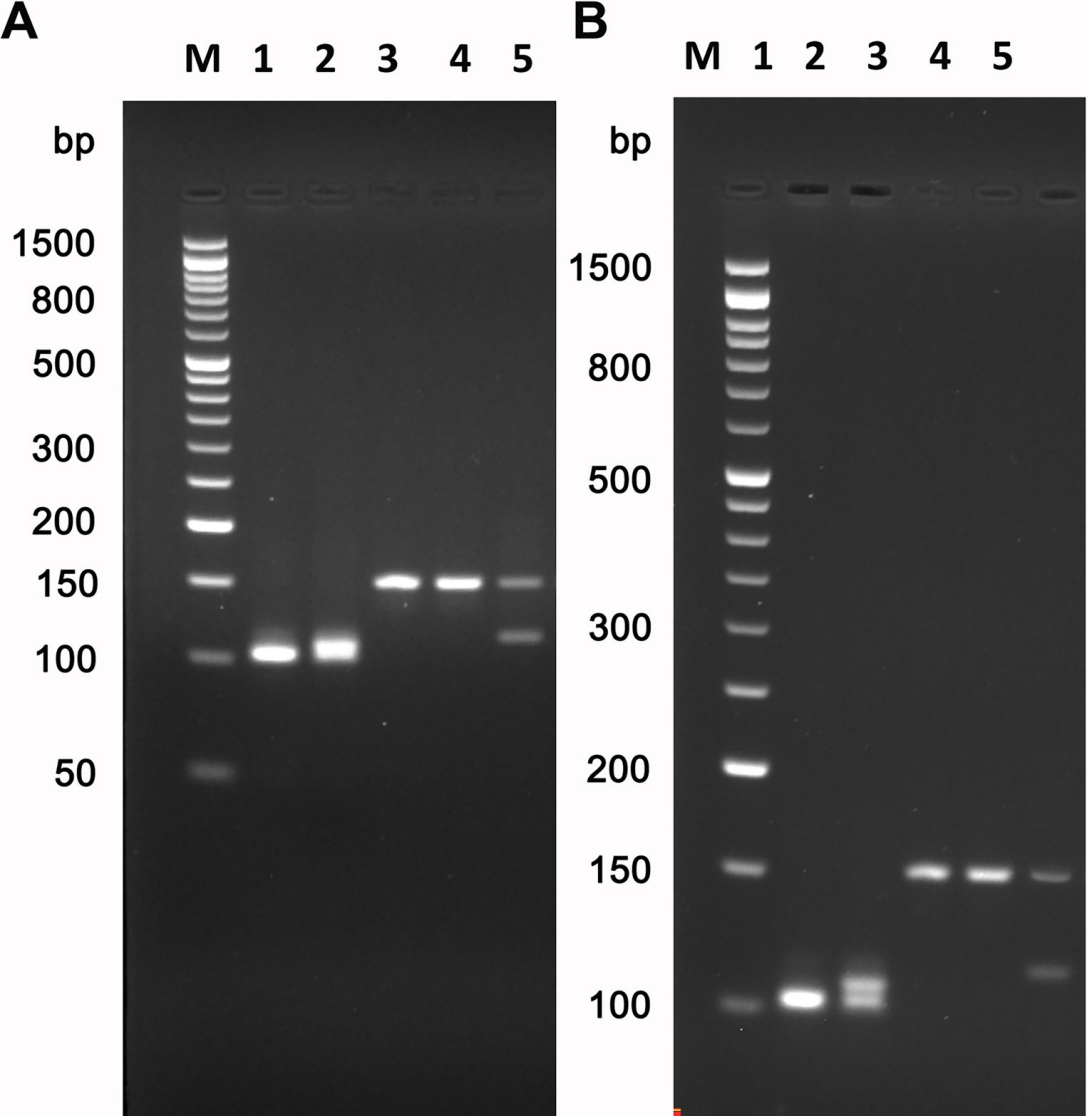
Agarose gel electrophoresis of amplicons produced in qPCR with *STS154/116* and *AMEL106/112* primers on modern DNA. **(**A) short electrophoretic run: in lanes 1 and 2 amplicons obtained with ***AMEL106/112*** on female and male DNA respectively; in lanes 3 and 4 amplicons obtained with ***STS154/116*** on female DNA; in lane 5 amplicons obtained with ***STS154/116*** on male DNA. (B) long electrophoretic run: in lanes 1 and 2 amplicons obtained with ***AMEL106/112*** on female and male DNA respectively; in lanes 3 and 4 amplicons obtained with ***STS154/116*** on female DNA; in lane 5 amplicons obtained with ***STS154/116*** on male DNA. M) SharpMass ™ 50 molecular weight marker—Ready-to-load DNA Ladder (Euroclone S.p.A). Agarose gels are 2.9% and stained with ethidium bromide as reported in Materials and Methods.

The presence of two amplicons in the male, which differ by a significant number of bp (38), and a single amplicon in the female could make this amplification strategy easier than that one currently used for sex determination with the amelogenin method (Fig 2).

From Fig 2 it is evident that through an electrophoretic analysis both primers allow to identify the sex, but the ***STS154/116*** primers are better performing. Fig 2A shows the results of a short electrophoretic run where the ***STS154/116*** primers give two bands in male individuals (Fig 2A lane 5) while the ***AMEL106/112*** primers give only one band on the male DNA (Fig 2A Lane 2). Only after a long electrophoretic run (Fig 2B) the ***AMEL106/112*** primers show distinct bands on the male DNA (Fig 2B lane 2).

The results obtained on modern DNA made it possible to identify the primers (***STS154/116***) capable of determining sex with the classical PCR method followed by an agarose gel or capillary electrophoresis. Therefore, these primers were tested in qPCR and PCR on male (S42, S43, S45, S46, S48) and female (S44, S47, S49, S50) ancient DNA samples. Unlike modern DNA, ***STS154 /116*** primers showed low accuracy on ancient DNA, since, as shown in S3 Table, no amplification was obtained in many tests; the expected amplicon is probably too large for the degraded aDNA.

### qPCR-duplex for sex determination

Sex determination using qPCR-duplex involves the co-amplification of two genes in a single reaction system and does not require any electrophoretic processing or separation after qPCR [26].

The thermal denaturation peaks of the amplicons predicted with the various primers, on the specific sequences, obtained with the in-silico analysis, as reported in the materials and methods section, allowed us to select and combine the best performing pairs of primers to be used in the qPCR-duplex (S3 Fig).

The qPCR-duplex was preliminarily performed on blood samples using 400 pg of male or female DNA as a template, in a 20 μl reaction system; these reactions allowed us to identify the pairs of best performing primers, their optimal concentration and the correspondence in number and size of the amplicons produced (Table 3).

**Fig 3:**
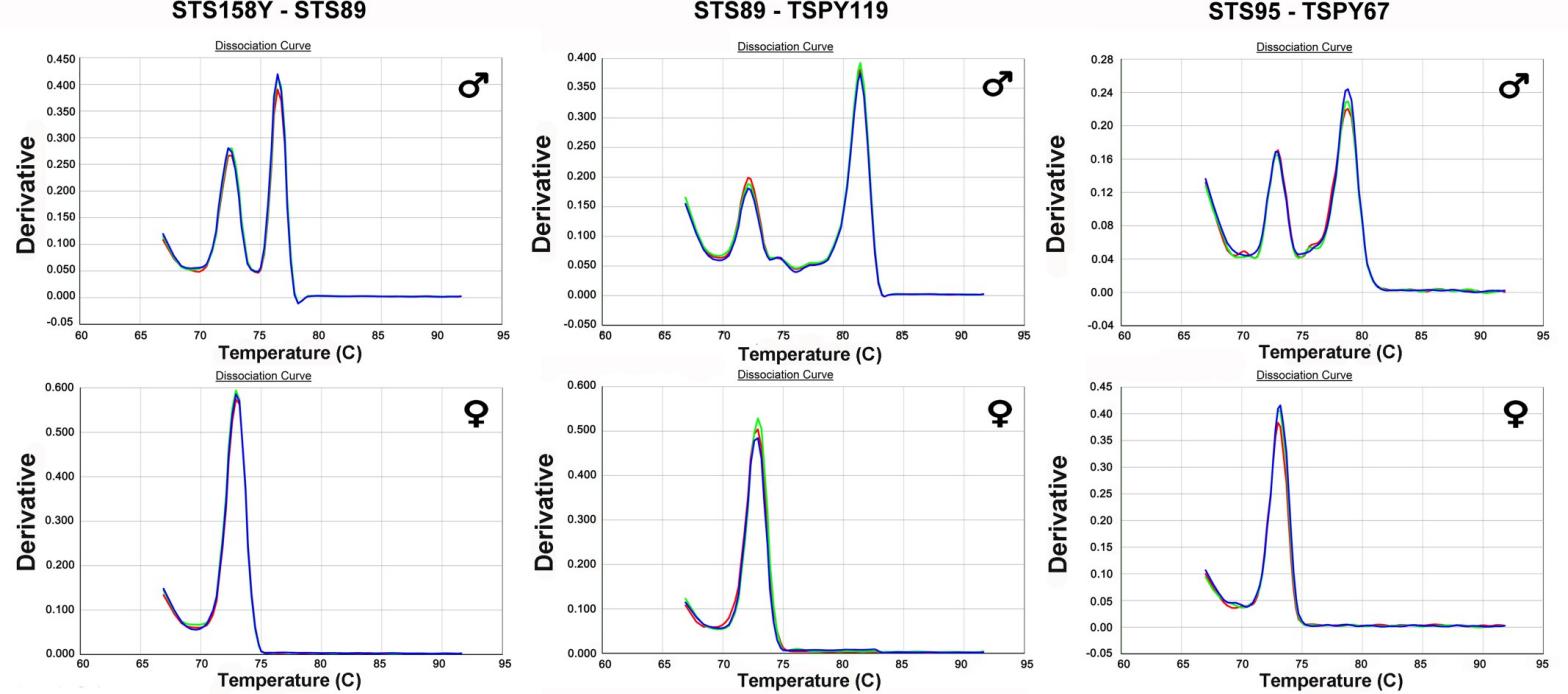
Thermal denaturation curves of amplification products in qPCR-duplex on modern DNA. Amplification products were obtained using 400 pg male (♂) and female (♀) DNA as a template in a 20 μl reaction system. The pairs of primers used were: ***STS158Y/STS89*** showing two amplification products, having Tm of 72.2°C and 76.5°C, and a single amplification product, having a Tm of 72.4°C, using male and female DNA respectively; ***STS89/TSPY119*** showing two amplification products, having Tm of 72.7°C and 81.5°C, and a single amplification product, having a Tm of 72.7°C, using male and female DNA respectively; ***STS95/TSPY67*** showing two amplification products, having Tm of 73.5 and 79.2°C, and a single amplification product, having a Tm of 72.7°C, using male and female DNA respectively.

Fig 3 shows the thermal denaturation curves of the amplicons obtained in qPCR-duplex with the pairs of primers selected on the male and female DNA extracted from the blood samples; the amplicons obtained confirm those expected from the in silico analysis.

The products obtained with the pair of primers ***STS158Y/STS89*** shows thermal denaturation curves that identify two peaks on the male DNA, having Tm 72.2° C and 76.5° C respectively and a single amplification product with Tm 72.4° C on the female DNA (Fig 3).

With the pair of primers ***STS89/TSPY119*,** two amplicons with male DNA having Tm 72.7° C and 81.5° C respectively and a single amplicon having Tm 72.7° C with female DNA were identified, (Fig 3).

Two amplicons with male DNA having Tm 73.5° C and 79.2° C respectively and a single amplicon having Tm 73.5° C with female DNA were also identified with the ***STS95/TSPY67*** pair of primers. (Fig 3).

The melting temperatures of the amplicons obtained in qPCR-duplex confirm those of the amplicons obtained in qPCR.

The qPCR-duplex method allowed us to determine the sex in blood samples only by analyzing the thermal denaturation curves, thanks to the use of two sex-specific markers.

**Fig 4:**
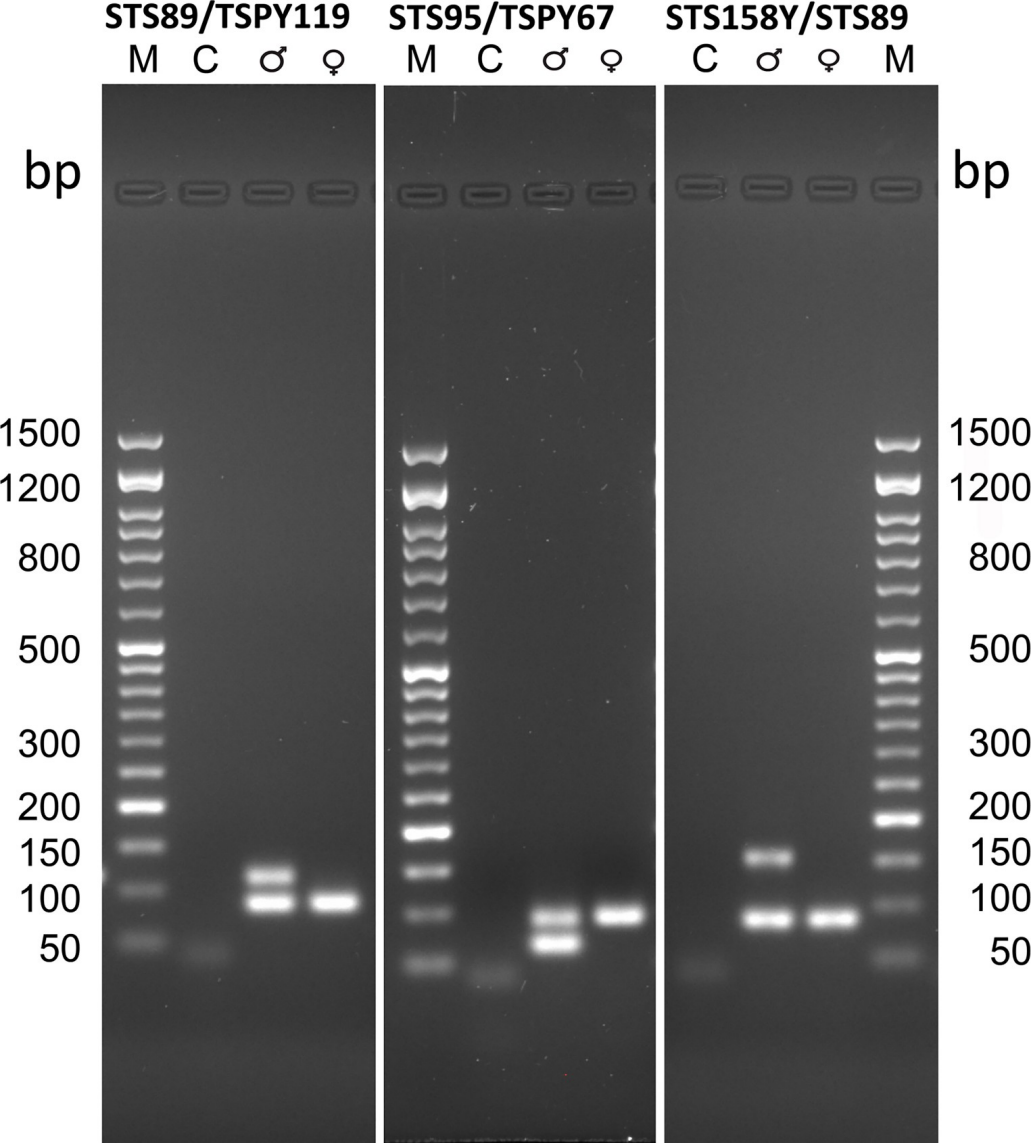
Agarose gel electrophoresis of the amplification products in qPCR-duplex on modern DNA. The three gels were loaded respectively with the qPCR-duplex reaction products obtained with the pairs of primers ***STS89/TSPY119***, ***STS95/TSPY67*** and ***STS158Y/STS89***: (C) control without DNA; lane 2 male DNA; lane 3 female DNA. M) SharpMass ™ 50 molecular weight marker—Ready-to-load DNA Ladder (Euroclone S.p.A). The gels were made with 2.2% agarose and stained with 0.5 μg / mL ethidium bromide.

Fig 4 shows the electrophoretic run of the amplicons produced in qPCR-duplex which confirms the results obtained from the analysis of the thermal denaturation curves as regards the number and size of the amplicons.

To assess whether the proposed method could be used for the determination of sex on ancient DNA, duplex qPCRs were performed on 9 DNA samples extracted from bone finds of different sex and from different eras, as described in the Materials and Methods section. The sex of these samples was unequivocally determined through an anthropometric and morphological analysis of the skeletons carried out by the archaeologists of the Department of Human Sciences of the University of L’Aquila and reported in Table 1.

Each sample was analyzed through two technical replicates, using 150 pg of DNA in the system as described in the Materials and Methods section.

**Fig 5:**
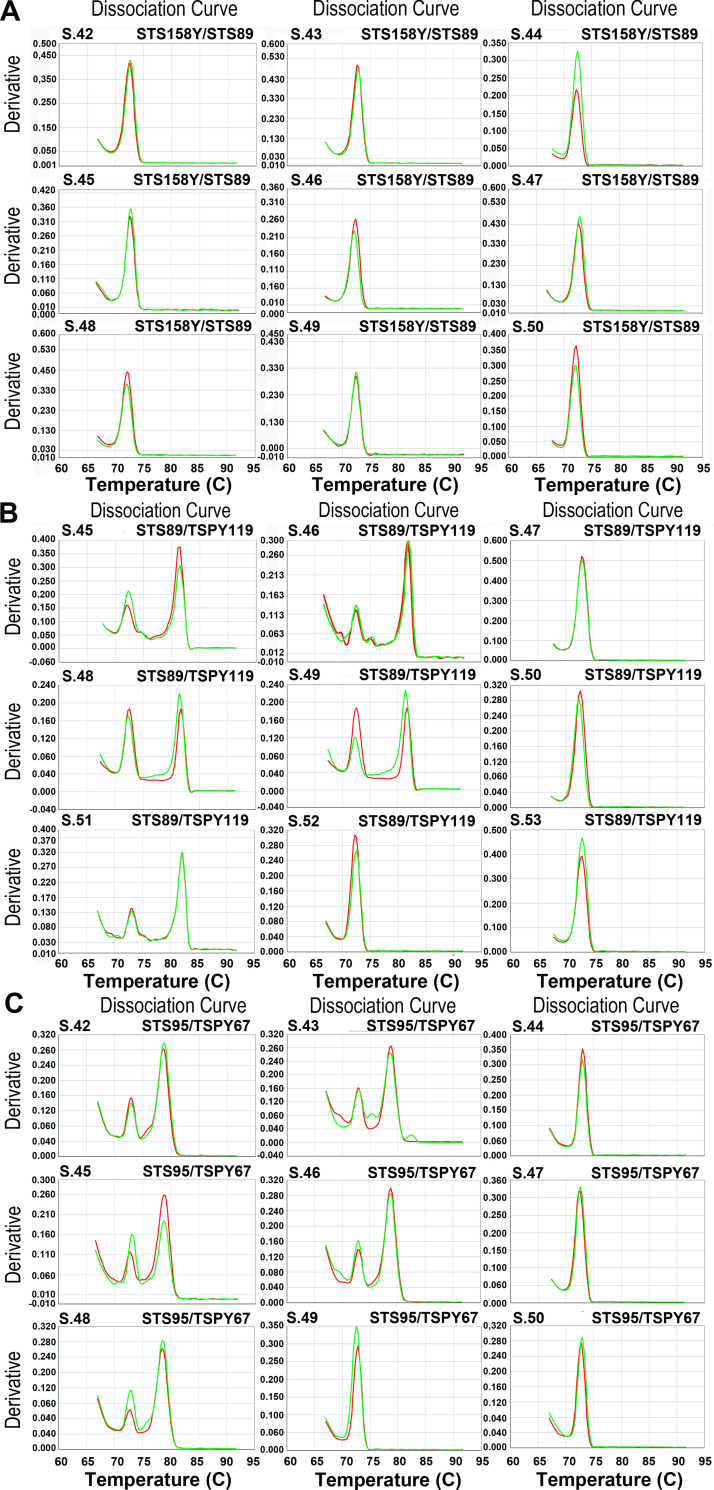
Thermal denaturation curves of the amplification products in qPCR-duplex on ancient DNA. Each extracted ancient DNA sample 150 pg was amplified in duplicate in qPCR with the pairs of primers ***STS158/STS89*** (A), ***STS89/TSPY119*** (B) and ***STS95/TSPY67*** (C). In B and C the male samples (S42, S43, S45, S46, S48) show two peaks while the female samples (S44, S47, S49, S50) only one peak. In A all samples show only one peak.

**Fig 6:**
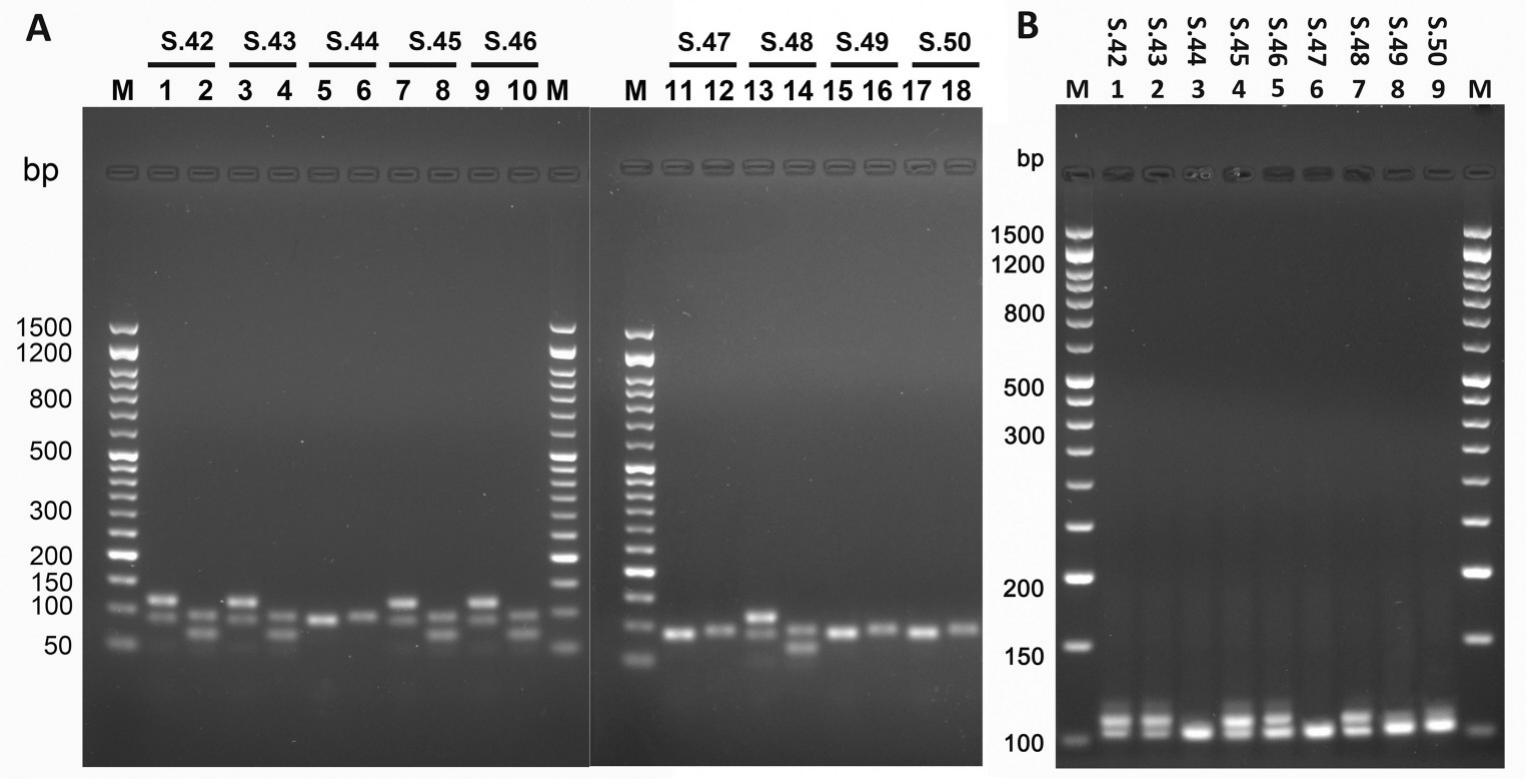
Agarose gel electrophoresis of the amplification products on ancient DNA. (A) The gels were loaded with the qPCR-duplex reaction products obtained with the pairs of primers ***STS89/TSPY119*, *STS95/TSPY67*** on male (S42, S43, S45, S46, S48) and female (S44, S47, S49, S50) DNA. In the channels (1,3,5,7,9,11,13,15,17), the products of qPCR with the pair of primers ***STS89/TSPY119*** are shown. In the channels (2,4,6,8,10,12,14,16) are reported the products of qPCR with the pair of primers ***STS95/TSPY67***. The gels were made with 2.2% agarose and stained with 0.5 μg/mL ethidium bromide. (B) Amplification fragments generated in qPCR on ancient DNA using the primers ***AMEL106/112***. The gels were made with 2.9% agarose and stained with 0.5 μg/mL ethidium bromide. M) marker of molecular weight, Sharp Mass ™ 50—Ready-to-load DNA Ladder (Euroclone S.p.A).

From Fig 5, which shows the thermal denaturation curves obtained with the pairs of primers ***STS158Y/STS89*, *STS89/TSPY119*** and ***STS95/TSPY67***, it can be seen that with the pairs of primers ***STS89/TSPY119*** and ***STS95/TSPY67***, two distinct amplicons in the male (S42, S43, S45, S46, S48) and one in the female (S44, S47, S49, S50) were obtained, as expected (Fig 5B and 5C); these results were also confirmed by the electrophoretic run (Fig 6A). Therefore, these pairs of primers could be able to clearly determine gender.

On the other hand, with the pair of primers ***STS158Y/STS89*** the expected results are not reached as only one amplicon is obtained in all samples, this can be attributed to the fact that ***STS158Y*** primers produce an amplicon that is too large for fragmented DNA such as aDNA (Fig 5A).

Fig 6B shows the electrophoretic run of the amplicons obtained in qPCR-duplex from the amplification of ancient DNA samples with ***AMEL106/112*** primers; these results confirm the ability of the pairs of primers ***STS89/TSPY119*** and ***STS95/TSPY67*** to determine sex (Figs 5B, 5C and 6A).

### Sensitivity and accuracy of the method

**Fig 7:**
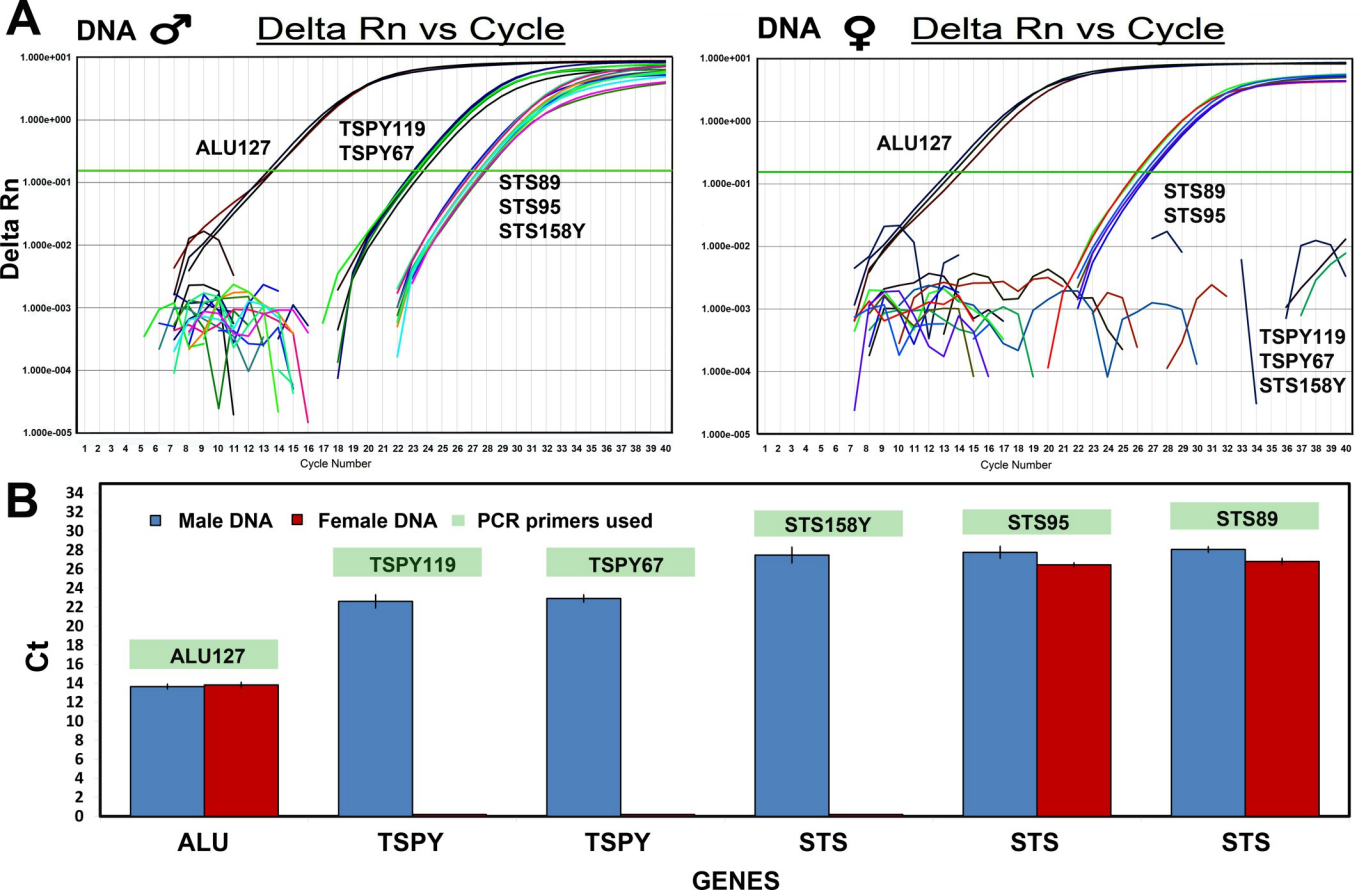
Sensitivity analysis in qPCR. **(**A) the amplification graphs show the fluorescence variation (Delta Rn) with respect to the number of cycles for the different amplicons obtained with the primers: ***ALU127***, ***TSPY119***, ***TSPY67***, ***STS89***, ***STS95***, ***STS158Y***; 400 pg of male and female DNA were used in the reaction system. B) the graph shows the mean Ct values of genes amplified using DNA from both sexes. The blue columns represent the male DNA, the red columns the female DNA.

The amplification plots and the graph in Fig 7 show that for the same concentration of DNA in the system, the TSPY gene has a lower Ct detection mean than the STS gene, so it has a higher copy/genome number as indicated in literature [51]. The TSPY gene is therefore more sensitive than the STS gene in sex determination and is preferred when the DNA concentration is low or when the DNA is degraded. The amplification plots identify the Alu consensus sequence as the most represented in the genome and sensitive enough to be used in qPCR to determine the concentration of the extracted DNA, when the sample concentration is low [52]. The two plots show that when a male DNA template is used, all genes are detected while with female DNA the TSPY gene did not produce amplifications.

Sensitivity was tested by amplifying DNA from blood samples with 1:10 dilution series up to 10 pg/ul in a 20 μl reaction system. The system is capable of amplifying the STS gene using 60 pg or more of DNA, while the TSPY gene with 20 pg or more of male or female DNA. For the pairs of primers used in qPCR-duplex, the sensitivity was 100 pg of DNA for the ***STS158Y/STS89*** pair of primers and 60 pg of DNA for the ***STS89/TSPY119*** and ***STS95/TSPY67*** pairs of primers in a 20 μl reaction system.

The qPCR in its classic and duplex configuration showed melting and Tm curves of the peaks always in agreement with what was expected in Table 3.

The use of ***STS154/116*** primers in PCR followed by electrophoretic run was always in agreement with the results obtained in qPCR and qPCR-duplex.

In blood samples, the accuracy with all tested primers capable of determining sex, both in qPCR and in qPCR-duplex, was found to be 100% (S2 Table). All the reaction products were subjected to electrophoretic running in order to verify accuracy, number and size of amplicons produced and the absence of non-specific amplification products.

The accuracy of the methods for sex determination was calculated also on the DNAa extracted from bone remains; amplicons produced in qPCR (***AMEL106/112***) and in qPCR-duplex (***STS158Y/STS89***, ***STS89/TSPY119*** and ***STS95/TSPY67***) were analyzed with electrophoretic run and denaturation curves respectively; from the results reported in Table 4 it is evident that, unlike in modern DNA samples, 100% accuracy is never obtained with ancient DNA, this is mainly attributable to the difficulty of extracting sufficiently intact DNA from the various samples, in fact some biological replicates B47 and B49 show an amplification defect. Moreover, unlike the results obtained on modern DNA, a different performance of the various primers on ancient DNA is evident.

**Table 4:** Accuracy of the qPCR-duplex and qPCR method on ancient DNA. The table shows the accuracy of the tests capable of identifying sex; amplicons obtained in qPCR-duplex and in PCR were analyzed through the melting curves and the electrophoretic run respectively. Three aDNA extractions were performed for each skeletal sample, the analysis was conducted with two technical replicates for each sample. In both qPCR-Duplex and PCR, 150 pg of DNA was used in a 20 μl reaction system. Gray cells amplification anomaly. NA) not amplified.

Samples	qPCR-duplex			PCR
	*STS89/STS158Y*	*STS89/TSPY119*	*STS95/TSPY67*	*AMEL106/112*
♂ S.42	89 bp	89–119 bp	95–67 bp	106–112 bp
	89 bp	89–119 bp	95–67 bp	106–112 bp
	89 bp	119 bp	95–67 bp	NA
♂ S.43	89 bp	89–119 bp	95–67 bp	106–112 bp
	89 bp	89–119 bp	95–67 bp	106–112 bp
	89 bp	89–119 bp	95–67 bp	NA
♂ S.45	89 bp	89–119 bp	95–67 bp	106–112 bp
	89 bp	89–119 bp	95–67 bp	106–112 bp
	89 bp	89–119 bp	95–67 bp	106–112 bp
♂ S.46	89 bp	89–119 bp	95–67 bp	106–112 bp
	89 bp	89–119 bp	95–67 bp	106–112 bp
	89–158 bp	89–119 bp	95–67 bp	106–112 bp
♂ S.48	89 bp	89–119 bp	95–67 bp	106–112 bp
	89 bp	89–119 bp	67 bp	106–112 bp
	89 bp	89–119 bp	95–67 bp	106–112 bp
♀ S.44	89 bp	89 bp	95 bp	106 bp
	NA	NA	NA	106 bp
	89 bp	89 bp	95 bp	106 bp
♀ B.47	89 bp	NA	95 bp	106 bp
	NA	89 bp	NA	NA
	NA	NA	95 bp	106 bp
♀ S.49	89 bp	89 bp	NA	NA
	89 bp	NA	95 bp	NA
	NA	NA	NA	106 bp
♀ S.50	89 bp	89 bp	95 bp	106 bp
	89 bp	89 bp	95 bp	106 bp
	89 bp	89 bp	95 bp	106 bp
Accuracy	33.3%	77.7%	81.4%	81.4%

In qPCR the ***AMEL106/112*** primers show an accuracy of 81.4%.

The ***STS158Y/STS89*** pair of primers shows 33.3% accuracy in qPCR-duplex, this low value on ancient DNA is attributable to the ***STS158Y*** primers since, in the same samples analyzed for qPCR, no amplification is observed in most cases (S3 Table and Fig 5). This could depend on the low number of copies per genome of the STS gene, and on the fact that ancient DNA is generally degraded, with fragments less than 200 bp [53]; it is difficult to obtain amplicons comparable in size to the fragments (158 bp).

The pairs of primers ***STS89/TSPY119*** and ***STS95/TSPY67*** show an accuracy of 77.7% and 81.4% respectively in qPCR-duplex, this high value is a consequence of the high reproducibility of the amplification obtained in qPCR with the primers ***STS89*, *STS95*, *TSPY119*** and ***TSY67*** (S3 Table).

## Conclusion

Ancient DNA, extracted from archaeological samples, is very fragmented and presents important modifications in its structure, a low extraction yield and amplification difficulty in PCR. In the existing literature, studies on human sex determination in degraded or ancient DNA by duplex qPCR use a single sex-specific marker [24,25].

In this work, we propose a duplex qPCR method, with subsequent analysis of the melting curves of the amplification products, which co-amplifies two sex-specific markers in a single reaction system; thus reducing the anomalies in sex determination.

The use of two markers in the human genome (steroid sulfatase (STS) and testis-specific Y-linked protein 1 (TSPY)) increases accuracy. In fact, through the use of SYBR Green within the analysis of the fusion curves, an unambiguous result is obtained as there is a clear difference in the shape of the melting peaks between male (2 peaks) and female (single peak) individuals, thus making this method more robust. Cross-contamination is also avoided because no processing after qPCR is required as is the case in other previously published methods [19].

The proposed method for sex determination was also found to be sensitive thanks to the use of the TSPY marker present in multiple copies in the human genome [30] and to the design of primers that generated small amplicons.

Primers built on the STS and TSPY genes that are used to amplify modern DNA, have excellent accuracy and good sensitivity with a detection limit of 60 pg and 20 pg in qPCR and 100 pg and 60 pg in qPCR-duplex respectively, confirming that the TSPY gene is more sensitive than the STS gene and therefore more suitable when DNA concentrations are low or when the DNA is degraded.

The accuracy of the method described in this paper has been confirmed on both modern and ancient DNA samples for which sex was known. All the results were compared with those obtained using the classic amelogenin method. In modern DNA, all the pairs of primers ***STS158Y / STS89*, *STS89 / TSPY119 and STS95 / TSPY67*** show 100% accuracy.

In ancient DNA the ***STS95 / TSPY67*** pair of primers shows an accuracy of 81.4% in qPCR-duplex, like the amelogenin test performed in PCR, this elevated value is a consequence of the high reproducibility of the amplification obtained in qPCR with primers ***STS95*** and ***TSY67***.

We also propose a PCR method based on the STS marker gene: the use of ***STS154/116***, which amplifies a fragment of 154 bp on the X allele and 116 bp on the Y allele, generates two amplicons in the male (154 bp and 116 bp) and a single amplicon in the female (154bp) easily distinguishable by electrophoresis; this method based on the deletion of 38 bp on STSY has proved to be, in modern DNA, an alternative to the amelogenin method [14].

Considering that sex typing is important in archaeology and anthropology for the study of societies, cultures and human activities of the past, the results obtained on ancient DNA are promising and worthy of being verified with samples from different eras. The duplex-qPCR method we have developed has the advantage of being more robust than previous methods; it is simple, inexpensive, fast and suitable for large-scale use for both modern and ancient DNA samples since it requires no additional work after the qPCR reaction.

## Supporting information

S1 Fig Strategy used for the choice of primers. (A) on the aligned sequences of the STS gene and of the pseudogene STSP1 the positions of the primers Forw_STS95_89, Rev_STS85 and Rev_STS95 are highlighted in blue, pink and green respectively. (B) the positions of the primers For_STS158Y and Rev_STS158Y are highlighted in yellow on the aligned sequences of the STS gene and of the pseudogene STSP1. (C) the positions of the primers For_STS154 / 116 and Rev_STS154 / 116 are highlighted in orange on the aligned sequences of the STS gene and of the pseudogene STSP1. (D) the positions of the primers FORW_1STS120 and REV_1STS120 are highlighted in gray on the aligned sequences of the STS gene and the pseudogene STSP1. (E) on the sequence of the TSPY gene the positions of the primers Forw_STS67 / 119, Rev_TSPY67 and Rev_TSPY119 are highlighted in light blue, purple and ocher respectively. For each pair of primers, the position on the sequence and the magnitude of the expected amplicon are shown.
(TIF)

S2 Fig Standard curves for the quantification of ancient DNA. The control DNA was amplified in qPCR with the primers ***ALU127*** and ***ALU50***. The standard curves, (A) ***Alu127*** and (B) ***Alu50*** were obtained with serial dilutions 1:10 from 29 ng/μl to 0.29 pg/μl, they show the Cts on the y axis and the log of the DNA concentration on the x axis. The slope, y-intercept, and correlation coefficient values are used to provide information on the performance of the reaction. (C) and (D) show plot, melting curves and electrophoretic run of the amplification products obtained with the ***ALU127*** and ***ALU50*** primers respectively. The amplification plots of ***Alu127*** and ***Alu50*** show an amplification in the control samples (NTC without template), due to environmental contamination.
(TIF)

S3 Fig In silico analysis. **(**A) Thermal denaturation peaks, obtained with in silico analysis, of the amplicons predicted with the various primers specific for the STS and TSPY genes. (B) Thermal denaturation peaks with different Tm side by side; possible pairs of primers to be used in qPCR-duplex.
(TIF)

S1 Table DNA concentration in samples of bone remains. Ancient DNA samples were amplified in qPCR with Alu127 and Alu50 primers. The table shows the mean values ± SD of the Ct and the corresponding concentration. The last column shows the mean ± SD of the two values of the concentrations.
(PDF)

S2 Table Assay for the accuracy of the qPCR-duplex method. The products of the amplification of 400 pg of DNA extracted from blood samples in qPCR-duplex, q-PCR and PCR are shown. The accuracy of the test was calculated on the ability of the primers pair to correctly identify the sex over the total number of tests performed. The qPCR-duplex method was validated and verified in qPCR by amplifying the ***STS*** and ***TSPY*** genes with the primers indicated in the table, the samples were verified by PCR with the ***STS154/116*** primers.
(PDF)

S3 Table Accuracy of the qPCR method on ancient DNA. The table shows the qPCR and PCR results with the primers selected to amplify the STS and TSPY genes. Three aDNA extractions were performed for each skeletal sample, the analysis was conducted with two technical replicates for each sample. In qPCR and PCR, 150 pg of DNA was used in a 20 μl reaction system. Gray cells anomaly in amplification; NA) not amplified.
(PDF)

S1 File Prime PCR ™ assay validation report. They are shown, for all constructed primers (STS158Y, STS89, STS154 / 116, STS95, STS120, TSPY67 and TSPY119) on the selected genes (STS—Steroid sulfatase and TSPY—testis specific protein Y-linked 1), in A) and B) the standard curves obtained by amplifying the DNA of the blood samples of males and females respectively, in C) Amplification plots and fusion curves of the amplicons obtained in qPCR and analysis of amplification products by electrophoresis. Standard curves were obtained with serial dilutions 1:10 from 1000 ng to 100 pg of DNA from blood samples. Each sample was analyzed in triplicate using 400 pg of male and female genomic DNA as a template in the reaction system.
(PDF)

S1 Raw images (PDF)
