# Supplementary figures and images for: A qPCR-duplex assay for sex determination in ancient DNA
Source: PLoS One. 2022 Jun 10;17(6):e0269913. doi: 10.1371/journal.pone.0269913 (PMC9187067; doi:10.1371/journal.pone.0269913)

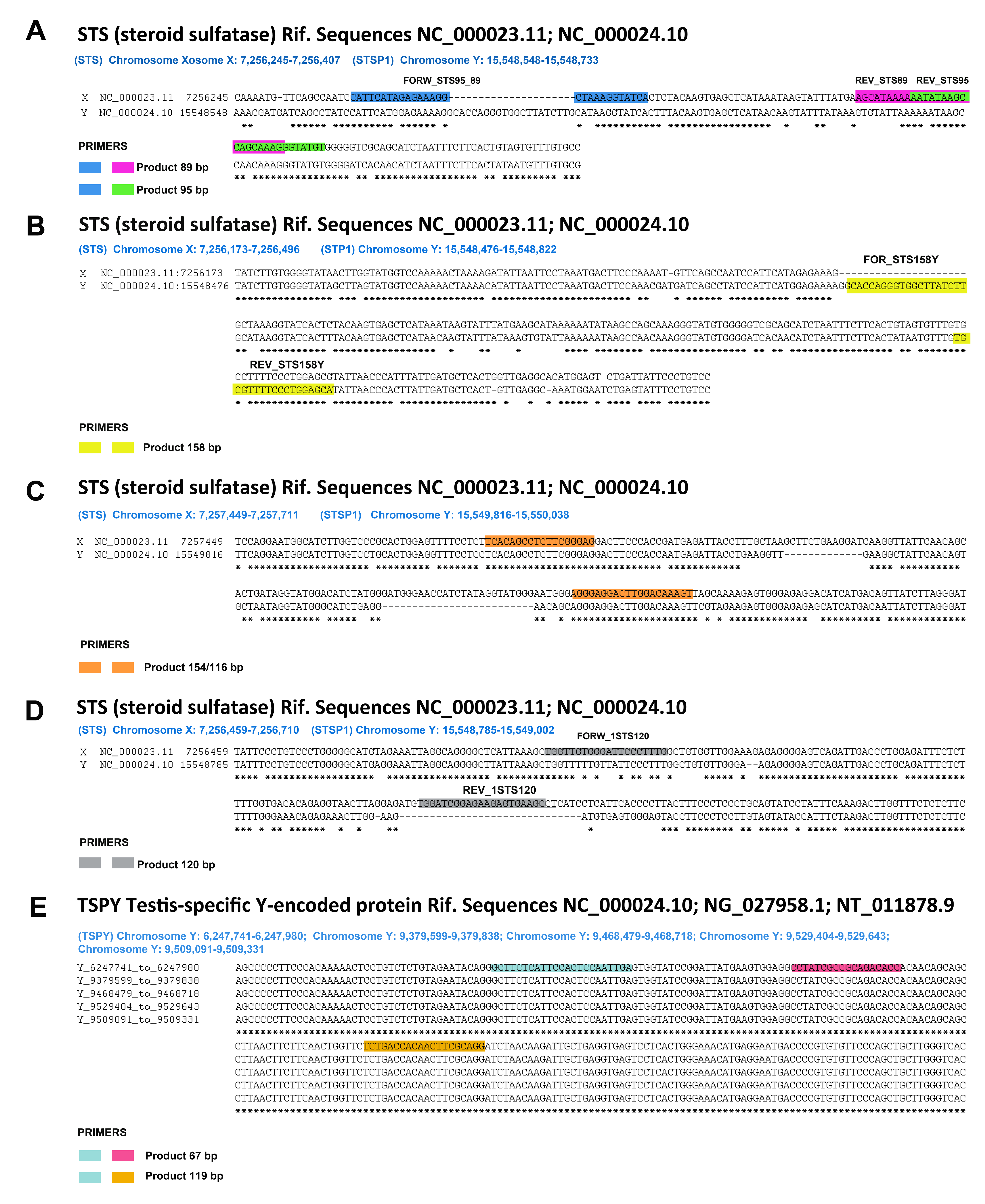

Supplement: S1 Fig — (A) on the aligned sequences of the STS gene and of the pseudogene STSP1 the positions of the primers Forw_STS95_89, Rev_STS85 and Rev_STS95 are highlighted in blue, pink and green respectively. (B) the positions of the primers For_STS158Y and Rev_STS158Y are highlighted in yellow on the aligned sequences of the STS gene and of the pseudogene STSP1. (C) the positions of the primers For_STS154 / 116 and Rev_STS154 / 116 are highlighted in orange on the aligned sequences of the STS gene and of the pseudogene STSP1. (D) the positions of the primers FORW_1STS120 and REV_1STS120 are highlighted in gray on the aligned sequences of the STS gene and the pseudogene STSP1. (E) on the sequence of the TSPY gene the positions of the primers Forw_STS67 / 119, Rev_TSPY67 and Rev_TSPY119 are highlighted in light blue, purple and ocher respectively. For each pair of primers, the position on the sequence and the magnitude of the expected amplicon are shown. (TIF) [file pone.0269913.s001.tif]

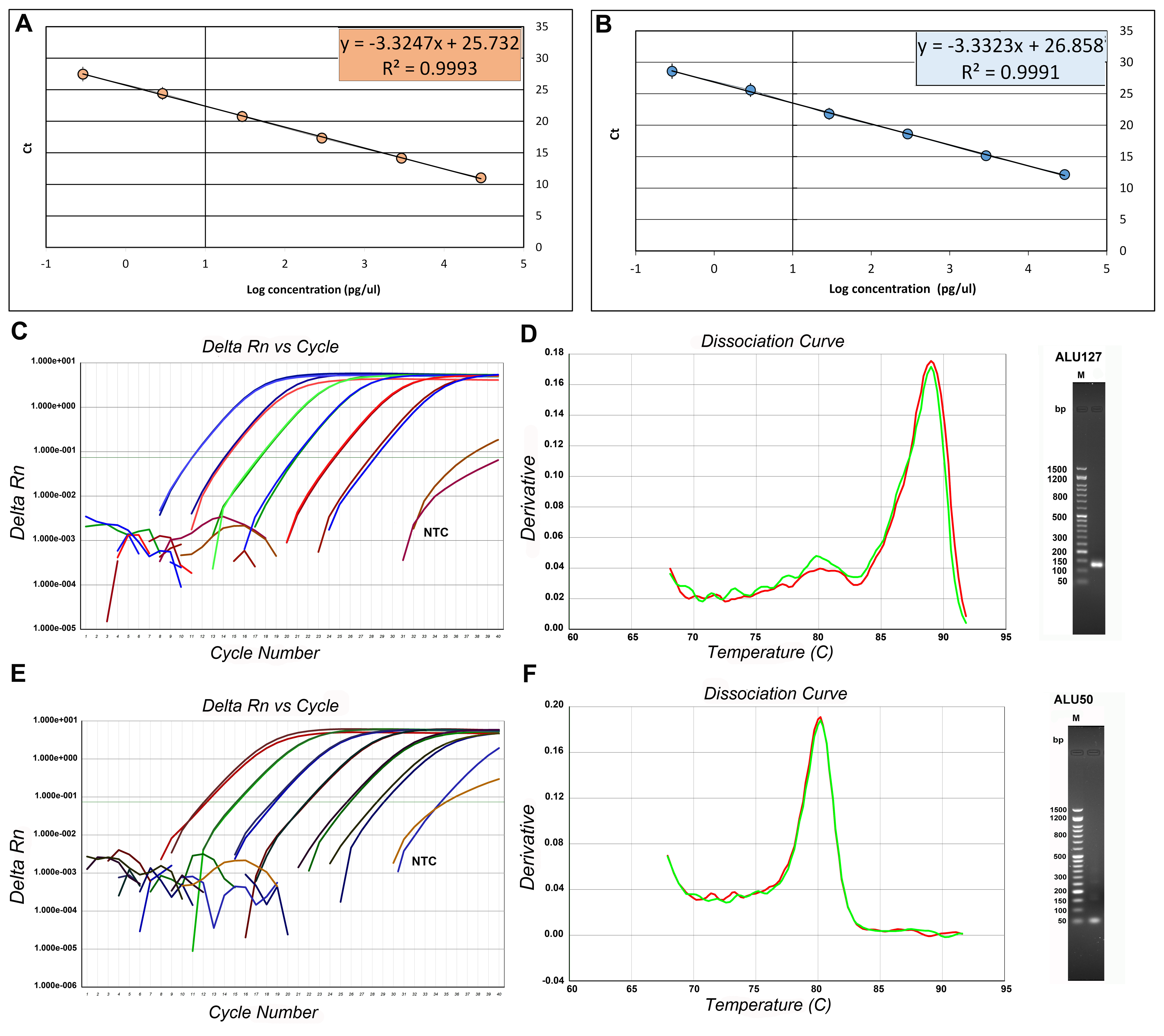

Supplement: S2 Fig — The control DNA was amplified in qPCR with the primers ALU127 and ALU50. The standard curves, (A) Alu127 and (B) Alu50 were obtained with serial dilutions 1:10 from 29 ng/μl to 0.29 pg/μl, they show the Cts on the y axis and the log of the DNA concentration on the x axis. The slope, y-intercept, and correlation coefficient values are used to provide information on the performance of the reaction. (C) and (D) show plot, melting curves and electrophoretic run of the amplification products obtained with the ALU127 and ALU50 primers respectively. The amplification plots of Alu127 and Alu50 show an amplification in the control samples (NTC without template), due to environmental contamination. (TIF) [file pone.0269913.s002.tif]

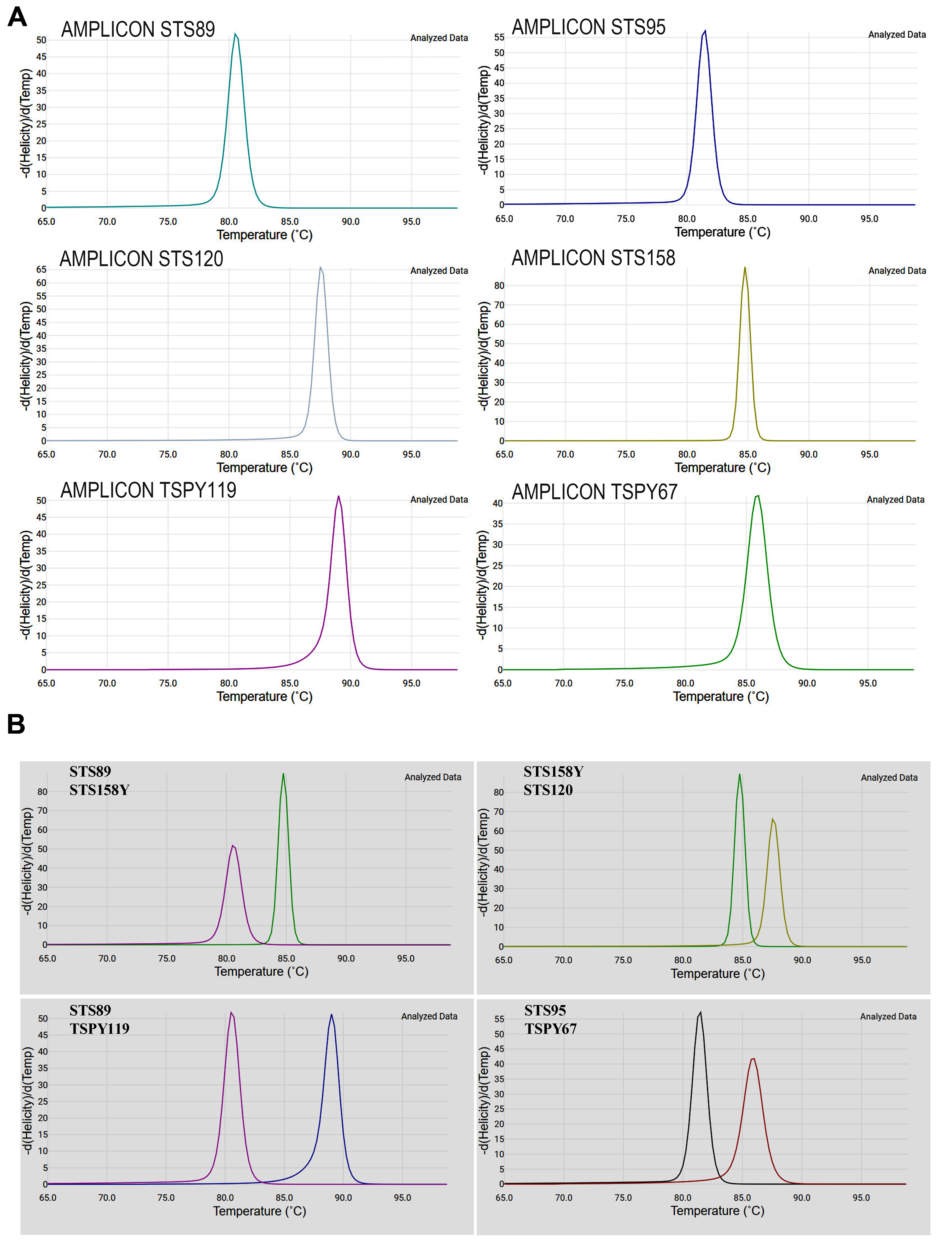

Supplement: S3 Fig — (A) Thermal denaturation peaks, obtained with in silico analysis, of the amplicons predicted with the various primers specific for the STS and TSPY genes. (B) Thermal denaturation peaks with different Tm side by side; possible pairs of primers to be used in qPCR-duplex. (TIF) [file pone.0269913.s003.tif]
