# Supplementary material for: A qPCR-duplex assay for sex determination in ancient DNA
Source: PLoS One. 2022 Jun 10;17(6):e0269913. doi: 10.1371/journal.pone.0269913 (PMC9187067; doi:10.1371/journal.pone.0269913)
Supplement: S1 Table — Ancient DNA samples were amplified in qPCR with Alu127 and Alu50 primers. The table shows the mean values ± SD of the Ct and the corresponding concentration. The last column shows the mean ± SD of the two values of the concentrations. (PDF) [file pone.0269913.s004.pdf]

S1 Table - DNA concentration in samples of bone remains. Ancient DNA samples were amplified in qPCR with Alu127 and Alu50 primers. The table shows the mean values ± SD of the Ct and the corresponding concentration. The last column shows the mean ± SD of the two values of the concentrations.

**Samples**	***ALU 127***		***ALU 50***		***ALU 127- ALU 50***
	**Ct mean values**	**pg/µl sample**	**Ct mean values**	**pg/µl sample**	**mean pg/ µl**
**B.42**	21.65±0.16	16.89±1.90	22.33±0.07	22.85±1.12	19.87±2.98
**B.43**	20.70±0.07	32.53±1.61	21.78±0.16	33.20±1.47	32.86±0.34
**B.44**	21.37±0.09	20.49±1.22	22.33±0.01	22.71±0.16	21.60±1.10
**B.45**	21.15±0.07	23.94±1.15	22.15±0.04	25.75±0.55	24.85±0.91
**B.46**	21.52±0.03	18.78±0.25	22.59±0.04	19.03±0.36	18.78±0.25
**B.47**	21.98±0.12	13.45±1.13	22.16±0.04	25.52±0.65	19.49±6.03
**B.48**	20.54±0.05	36.51±1.29	21.47±0.01	41.12±0.16	38.81±2.30
**B.49**	20.95±0.09	27.39±1.61	21.89±0.02	30.81±0.31	29.10±1.71
**B.50**	21.10±0.04	24.76±0.66	22.01±0.03	28.33±0.22	26.55±1.79
