# Supplementary material for: A qPCR-duplex assay for sex determination in ancient DNA
Source: PLoS One. 2022 Jun 10;17(6):e0269913. doi: 10.1371/journal.pone.0269913 (PMC9187067; doi:10.1371/journal.pone.0269913)
Supplement: S2 Table — The products of the amplification of 400 pg of DNA extracted from blood samples in qPCR-duplex, q-PCR and PCR are shown. The accuracy of the test was calculated on the ability of the primers pair to correctly identify the sex over the total number of tests performed. The qPCR-duplex method was validated and verified in qPCR by amplifying the STS and TSPY genes with the primers indicated in the table, the samples were verified by PCR with the STS154/116 primers. (PDF) [file pone.0269913.s005.pdf]

S2 Table - Assay for the accuracy of the qPCR-duplex method. The products of the amplification of 400 pg of DNA extracted from blood samples in qPCR-duplex, q-PCR and PCR are shown. The accuracy of the test was calculated on the ability of the primers pair to correctly identify the sex over the total number of tests performed. The qPCR-duplex method was validated and verified in qPCR by amplifying the ***STS*** and ***TSPY*** genes with the primers indicated in the table, the samples were verified by PCR with the ***STS154/116*** primers.

Samples	qPCR-duplex			qPCR					PCR
	STS158Y/STS89	***STS89/TSPY119***	***STS95/TSPY67***	***STS158Y***	***STS89***	***STS95***	***TSPY119***	***TPY67***	***STS154/116***
1 ♂	158-89 bp	89-119 bp	95-67 bp	158 bp	89 bp	95 bp	119 bp	67 bp	154 -116 bp
	158-89 bp	89-119 bp	95-67 bp	158 bp	89 bp	95 bp	119 bp	67 bp	154 -116 bp
	158-89 bp	89-119 bp	95-67 bp	158 bp	89 bp	95 bp	119 bp	67 bp	154 -116 bp
2 ♂	158-89 bp	89-119 bp	95-67 bp	158 bp	89 bp	95 bp	119 bp	67 bp	154 -116 bp
	158-89 bp	89-119 bp	95-67 bp	158 bp	89 bp	95 bp	119 bp	67 bp	154 -116 bp
	158-89 bp	89-119 bp	95-67 bp	158 bp	89 bp	95 bp	119 bp	67 bp	154 -116 bp
4 ♂	158-89 bp	89-119 bp	95-67 bp	158 bp	89 bp	95 bp	119 bp	67 bp	154 -116 bp
	158-89 bp	89-119 bp	95-67 bp	158 bp	89 bp	95 bp	119 bp	67 bp	154 -116 bp
	158-89 bp	89-119 bp	95-67 bp	158 bp	89 bp	95 bp	119 bp	67 bp	154 -116 bp
7 ♂	158-89 bp	89-119 bp	95-67 bp	158 bp	89 bp	95 bp	119 bp	67 bp	154 -116 bp
	158-89 bp	89-119 bp	95-67 bp	158 bp	89 bp	95 bp	119 bp	67 bp	154 -116 bp
	158-89 bp	89-119 bp	95-67 bp	158 bp	89 bp	95 bp	119 bp	67 bp	154 -116 bp
8 ♂	158-89 bp	89-119 bp	95-67 bp	158 bp	89 bp	95 bp	119 bp	67 bp	154 -116 bp
	158-89 bp	89-119 bp	95-67 bp	158 bp	89 bp	95 bp	119 bp	67 bp	154 -116 bp
	158-89 bp	89-119 bp	95-67 bp	158 bp	89 bp	95 bp	119 bp	67 bp	154 -116 bp
10 ♂	158-89 bp	89-119 bp	95-67 bp	158 bp	89 bp	95 bp	119 bp	67 bp	154 -116 bp
	158-89 bp	89-119 bp	95-67 bp	158 bp	89 bp	95 bp	119 bp	67 bp	154 -116 bp
	158-89 bp	89-119 bp	95-67 bp	158 bp	89 bp	95 bp	119 bp	67 bp	154 -116 bp
11 ♂	158-89 bp	89-119 bp	95-67 bp	158 bp	89 bp	95 bp	119 bp	67 bp	154 -116 bp
	158-89 bp	89-119 bp	95-67 bp	158 bp	89 bp	95 bp	119 bp	67 bp	154 -116 bp
	158-89 bp	89-119 bp	95-67 bp	158 bp	89 bp	95 bp	119 bp	67 bp	154 -116 bp
12 ♂	158-89 bp	89-119 bp	95-67 bp	158 bp	89 bp	95 bp	119 bp	67 bp	154 -116 bp
	158-89 bp	89-119 bp	95-67 bp	158 bp	89 bp	95 bp	119 bp	67 bp	154 -116 bp
	158-89 bp	89-119 bp	95-67 bp	158 bp	89 bp	95 bp	119 bp	67 bp	154 -116 bp
15 ♂	158-89 bp	89-119 bp	95-67 bp	158 bp	89 bp	95 bp	119 bp	67 bp	154 -116 bp
	158-89 bp	89-119 bp	95-67 bp	158 bp	89 bp	95 bp	119 bp	67 bp	154 -116 bp
	158-89 bp	89-119 bp	95-67 bp	158 bp	89 bp	95 bp	119 bp	67 bp	154 -116 bp
16 ♂	158-89 bp	89-119 bp	95-67 bp	158 bp	89 bp	95 bp	119 bp	67 bp	154 -116 bp
	158-89 bp	89-119 bp	95-67 bp	158 bp	89 bp	95 bp	119 bp	67 bp	154 -116 bp
	158-89 bp	89-119 bp	95-67 bp	158 bp	89 bp	95 bp	119 bp	67 bp	154 -116 bp
3 ♀	89 bp	89 bp	95 bp		89 bp	95 bp			154 bp
	89 bp	89 bp	95 bp		89 bp	95 bp			154 bp
	89 bp	89 bp	95 bp		89 bp	95 bp			154 bp
5 ♀	89 bp	89 bp	95 bp		89 bp	95 bp			154 bp
	89 bp	89 bp	95 bp		89 bp	95 bp			154 bp
	89 bp	89 bp	95 bp		89 bp	95 bp			154 bp
6 ♀	89 bp	89 bp	95 bp		89 bp	95 bp			154 bp
	89 bp	89 bp	95 bp		89 bp	95 bp			154 bp
	89 bp	89 bp	95 bp		89 bp	95 bp			154 bp
9 ♀	89 bp	89 bp	95 bp		89 bp	95 bp			154 bp
	89 bp	89 bp	95 bp		89 bp	95 bp			154 bp
	89 bp	89 bp	95 bp		89 bp	95 bp			154 bp
13 ♀	89 bp	89 bp	95 bp		89 bp	95 bp			154 bp
	89 bp	89 bp	95 bp		89 bp	95 bp			154 bp
	89 bp	89 bp	95 bp		89 bp	95 bp			154 bp
14 ♀	89 bp	89 bp	95 bp		89 bp	95 bp			154 bp
	89 bp	89 bp	95 bp		89 bp	95 bp			154 bp
	89 bp	89 bp	95 bp		89 bp	95 bp			154 bp
17 ♀	89 bp	89 bp	95 bp		89 bp	95 bp			154 bp .
	89 bp	89 bp	95 bp		89 bp	95 bp			154 bp
	89 bp	89 bp	95 bp		89 bp	95 bp			154 bp
18♀	89 bp	89 bp	95 bp		89 bp	95 bp			154 bp
	89 bp	89 bp	95 bp		89 bp	95 bp			154 bp
	89 bp	89 bp	95 bp		89 bp	95 bp			154 bp
Accuracy	100%	100%	100%						100%
