# Supplementary material for: A qPCR-duplex assay for sex determination in ancient DNA
Source: PLoS One. 2022 Jun 10;17(6):e0269913. doi: 10.1371/journal.pone.0269913 (PMC9187067; doi:10.1371/journal.pone.0269913)
Supplement: S3 Table — The table shows the qPCR and PCR results with the primers selected to amplify the STS and TSPY genes. Three aDNA extractions were performed for each skeletal sample, the analysis was conducted with two technical replicates for each sample. In qPCR and PCR, 150 pg of DNA was used in a 20 μl reaction system. Gray cells anomaly in amplification; NA) not amplified. (PDF) [file pone.0269913.s006.pdf]

S3 Table - Accuracy of the qPCR method on ancient DNA. The table shows the qPCR and PCR results with the primers selected to amplify the STS and TSPY genes. Three aDNA extractions were performed for each skeletal sample, the analysis was conducted with two technical replicates for each sample. In qPCR and PCR, 150 pg of DNA was used in a 20 µl reaction system. Gray cells anomaly in amplification; NA) not amplified.

Samples	qPCR					PCR	
	STS158Y	***STS89***	***STS95***	***TSPY119***	***TSPY67***	***STS154/116***	***AMEL106/112***
♂ S.42	NA	89 bp	95 bp	119 bp	67bp	154-116 bp	106-112 bp
	NA	NA	NA	119 bp	67bp	NA	106-112 bp
	NA	89 bp	95 bp	119 bp	67bp	154-116 bp	NA
♂ S.43	NA	89 bp	95 bp	NA	NA	154-116 bp	106-112 bp
	158 bp	89 bp	NA	119 bp	67bp	NA	106-112 bp
	NA	89 bp	95 bp	119 bp	67bp	NA	NA
♂ S.45	NA	89 bp	95 bp	119 bp	67bp	154-116 bp	106-112 bp
	NA	89 bp	95 bp	119 bp	67bp	NA	106-112 bp
	NA	NA	95 bp	119 bp	67bp	154-116 bp	106-112 bp
♂ S.46	NA	89 bp	95 bp	NA	67bp	NA	106-112 bp
	NA	89 bp	95 bp	NA	NA	154-116 bp	106-112 bp
	158 bp	89 bp	95 bp	119 bp	67bp	NA	106-112 bp
♂ S.48	NA	89 bp	95 bp	119 bp	NA	154-116 bl	106-112 bp
	158 bp	89 bp	NA	119 bp	67bp	154-116 bp	106-112 bp
	158 bp	89 bp	95 bp	119 bp	67bp	154-116 bp	106-112 bp
♀ S.44	NA	89 bp	95 bp	NA	NA	NA	106 bp
	NA	NA	95 bp	NA	NA	154 bp	106 bp
	NA	89 bp	95 bp	NA	NA	154 bp	106 bp
♀ S.47	NA	89 bp	NA	NA	NA	NA	106 bp
	NA	89 bp	NA	NA	NA	NA	NA
	NA	NA	95 bp	NA	NA	154 bp	106 bp
♀ S.49	NA	89 bp	95 bp	NA	NA	154 bp	NA
	NA	89 bp	NA	NA	NA	NA	NA
	NA	89 bp	95 bp	NA	NA	154 bp	106 bp
♀ S.50	NA	89 bp	95 bp	NA	NA	NA	106 bp
	NA	89 bp	95 bp	NA	NA	NA	106 bp
	NA	89 bp	95 bp	NA	NA	154 bp	106 bp
Accuracy		85.19 %	81.4 %			55.5 %	81.4 %
