# Supplementary material for: A qPCR-duplex assay for sex determination in ancient DNA
Source: PLoS One. 2022 Jun 10;17(6):e0269913. doi: 10.1371/journal.pone.0269913 (PMC9187067; doi:10.1371/journal.pone.0269913)
Supplement: S1 File — They are shown, for all constructed primers (STS158Y, STS89, STS154 / 116, STS95, STS120, TSPY67 and TSPY119) on the selected genes (STS—Steroid sulfatase and TSPY—testis specific protein Y-linked 1), in A) and B) the standard curves obtained by amplifying the DNA of the blood samples of males and females respectively, in C) Amplification plots and fusion curves of the amplicons obtained in qPCR and analysis of amplification products by electrophoresis. Standard curves were obtained with serial dilutions 1:10 from 1000 ng to 100 pg of DNA from blood samples. Each sample was analyzed in triplicate using 400 pg of male and female genomic DNA as a template in the reaction system. (PDF) [file pone.0269913.s007.pdf]

STS GENE – Steroid Sulfatase

STS158Y Primers

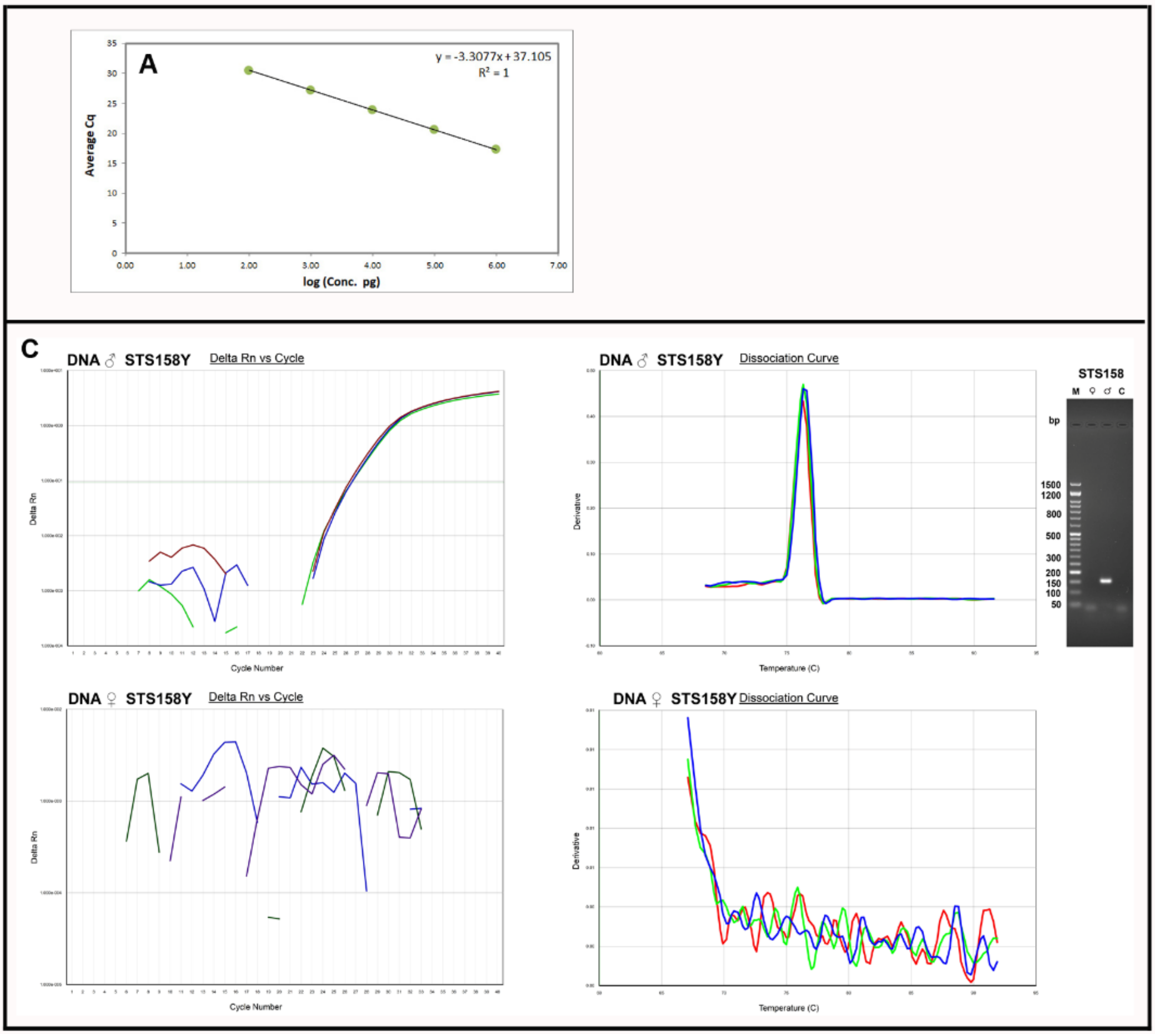

STS89 Primers

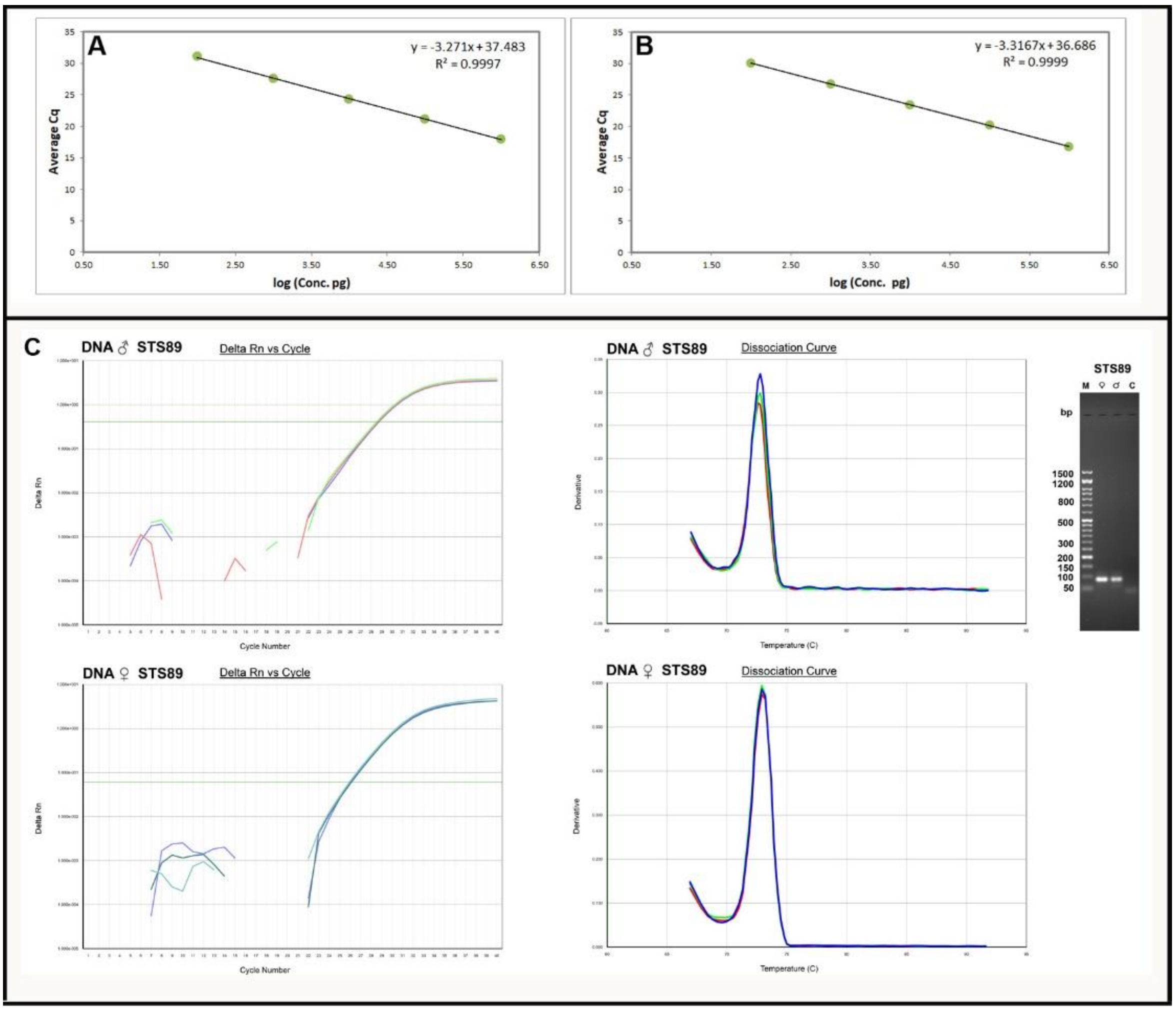

STS154-116 Primers

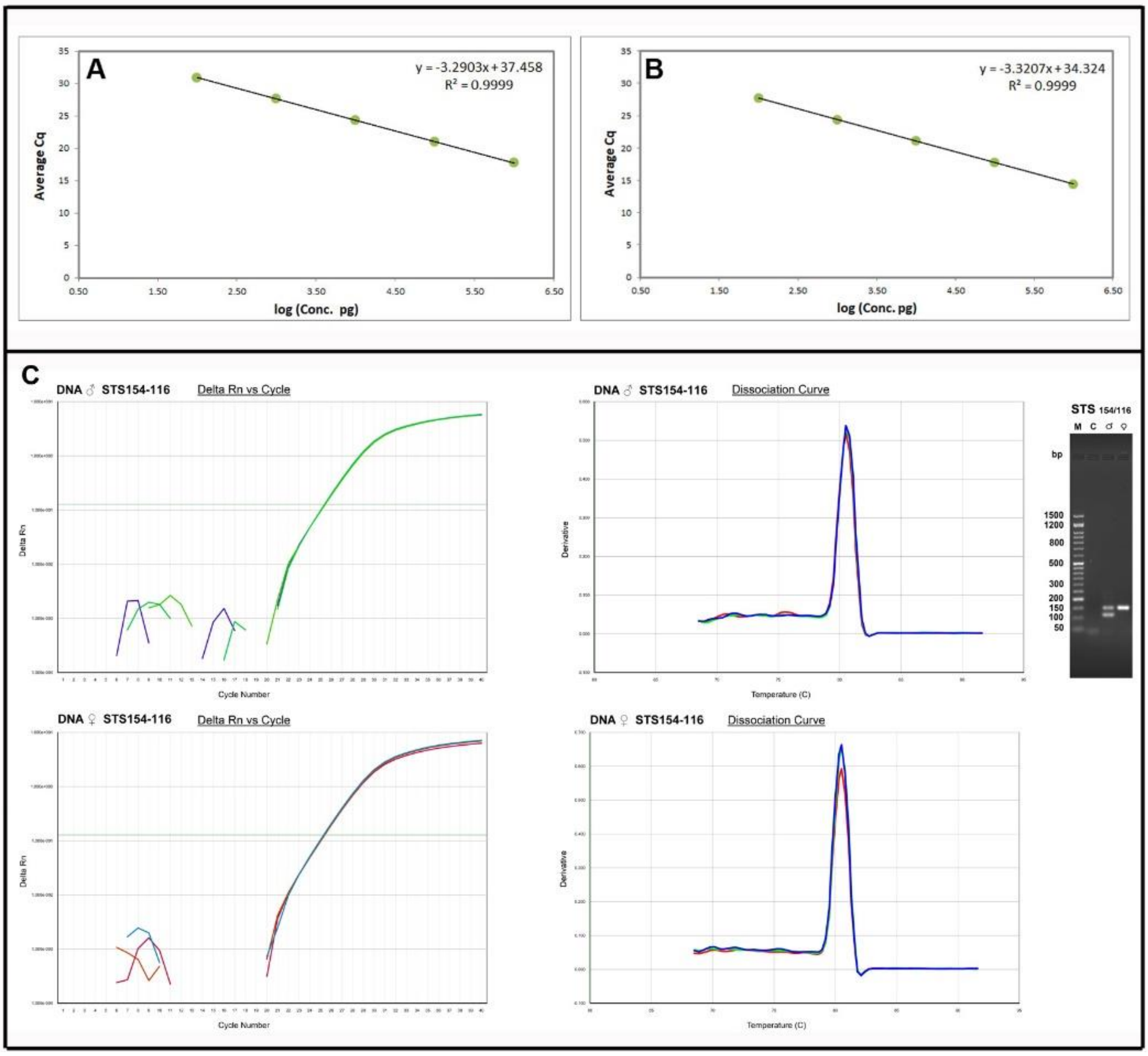

STS95 Primers

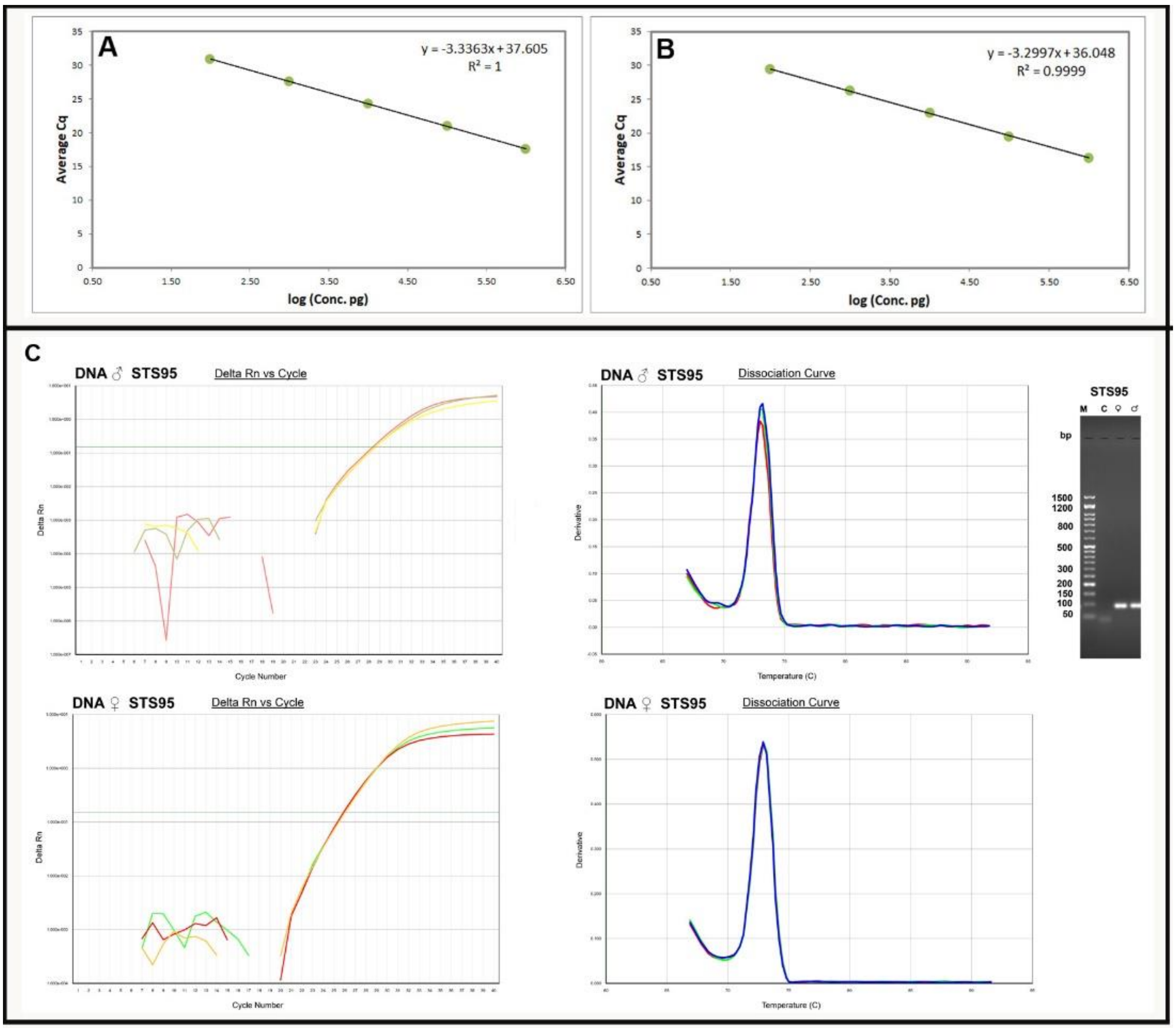

STS120 Primers

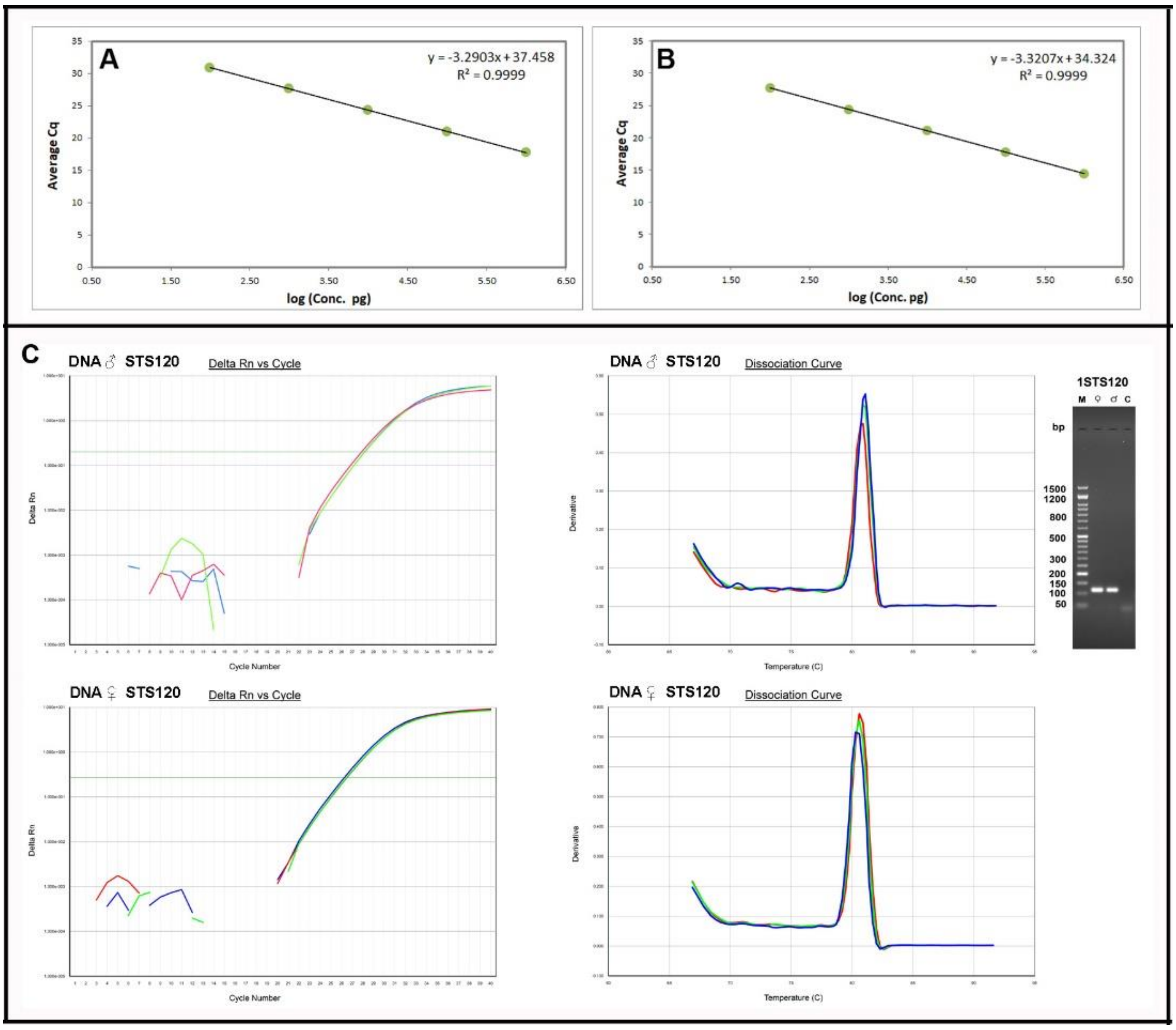

TSPY GENE - testis specific protein Y-linked 1

TSPY67 Primers

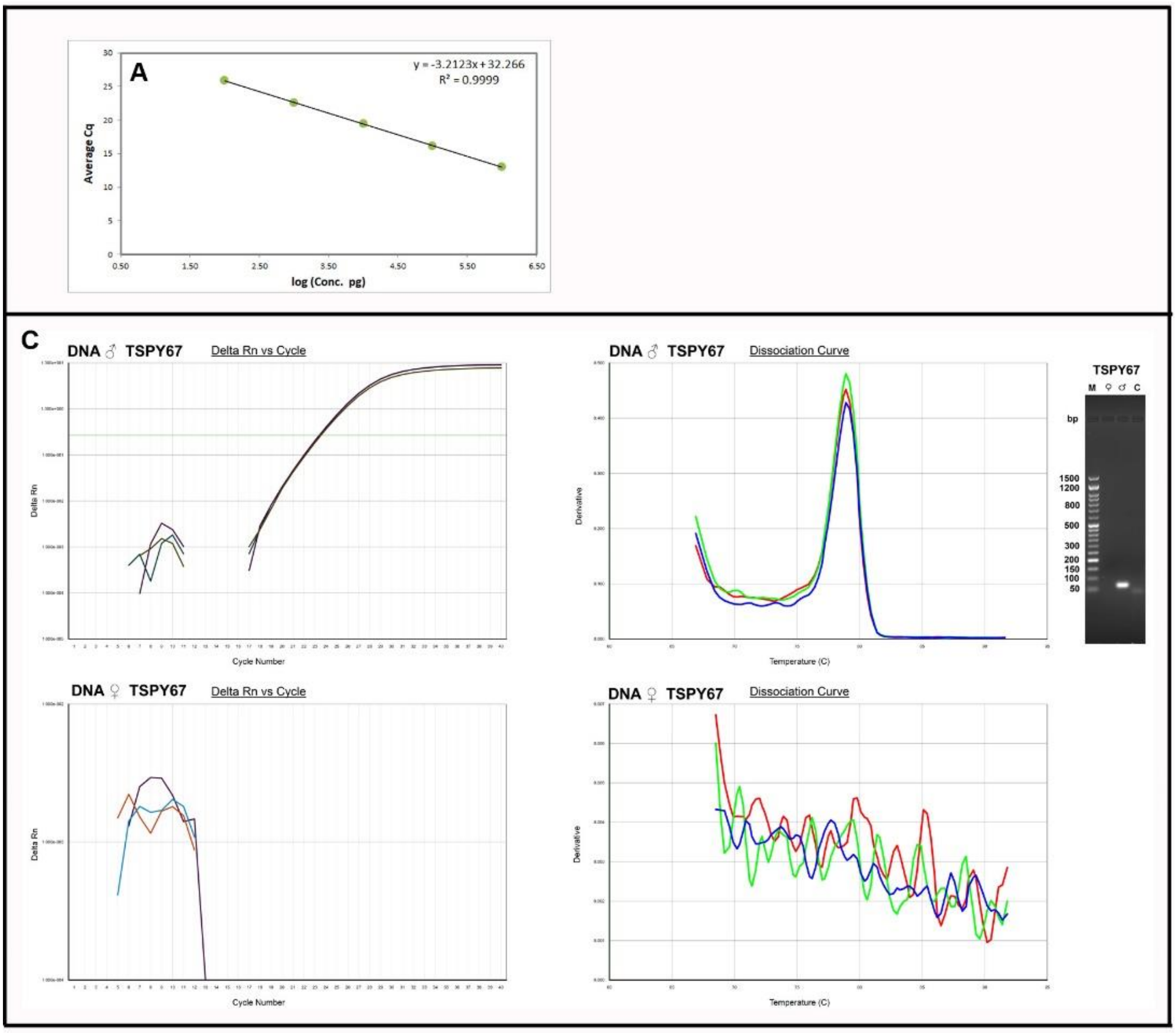

TSPY119 Primers

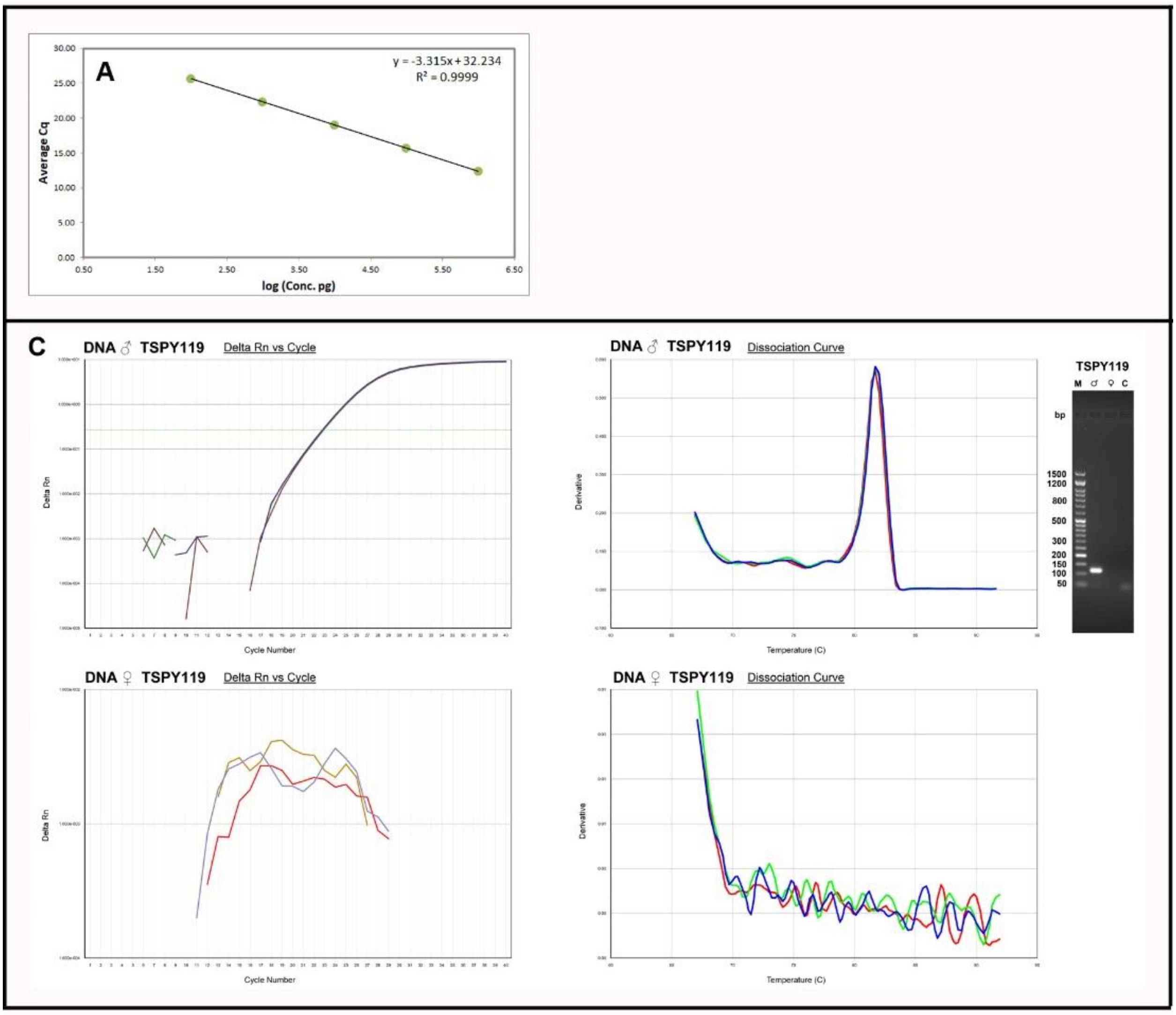

S1 File - Prime PCR ™ Assay Validation Report.

They are shown, for all constructed primers (***STS158Y, STS89, STS154 / 116, STS95, STS120, TSPY67 and TSPY119***) on the selected genes (STS - Steroid sulfatase and TSPY - testis specific protein Y-linked 1), in A) and B) the standard curves obtained by amplifying the DNA of the blood samples of males and females respectively, in C) Amplification plots and fusion curves of the amplicons obtained in qPCR and analysis of amplification products by electrophoresis.

Standard curves were obtained with serial dilutions 1:10 from 1000 ng to 100 pg of DNA from blood samples. Each sample was analyzed in triplicate using 400 pg of male and female genomic DNA as a template in the reaction system.
