# Supplementary material for: A qPCR-duplex assay for sex determination in ancient DNA
Source: PLoS One. 2022 Jun 10;17(6):e0269913. doi: 10.1371/journal.pone.0269913 (PMC9187067; doi:10.1371/journal.pone.0269913)
Supplement: S1 Raw images — (PDF) [file pone.0269913.s008.pdf]

S1_raw_images

A qPCR-DUPLEX ASSAY FOR SEX DETERMINATION IN ANCIENT DNA

Anna[1] Poma, Patrizia[1] Cesare, Antonella[1] Bonfigli, Anna Rita[1] Volpe, Sabrina[1] Colafarina, Giulia[1] Vecchiotti, Alfonso[2] Forgione, Osvaldo[1*] Zarivi.

[1]Department of Life, Health and Environmental Sciences, University of L'Aquila, L'Aquila, Italy

[2]Department of Human Studies, University of L'Aquila, L'Aquila, Italy

*corresponding authors

E-mail address: osvaldo.zarivi@univaq.it (O. Zarivi)

S1 raw images
Figure 1

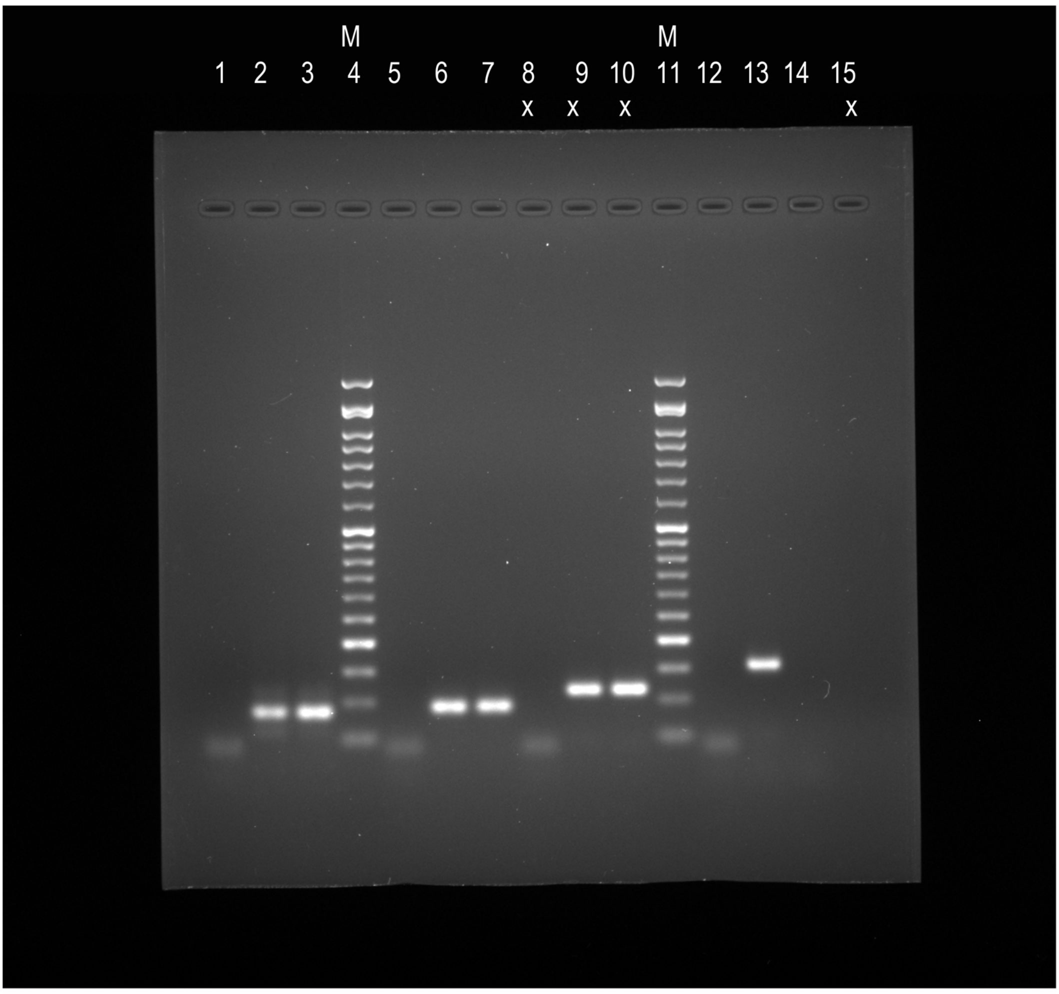

This image was partially used to generate the panel of figure 1.
Agarose gel electrophoresis of amplicons generated in qPCR using primers STS89 (lane 1, 2, 3 overturned horizontally), STS95 (lane 5, 6, 7) and STS158Y (lane 12,13,14) with modern human male and female DNA.
M (lane 4,11) SharpMass™ 50 molecular weight marker - Ready-to-load DNA Ladder (Euroclone S.p.A) DNA fragments: 50,100,150,200, 250, 300, 350,400, 450,500, 600, 700,800, 900, 1000, 1200 and 1500 bp. Agarose gels are 2.2% and stained with ethidium bromide as reported in Materials and Methods. Gels were acquired with the Gel Doc XR+Gel Documentation System (Bio-Rad).

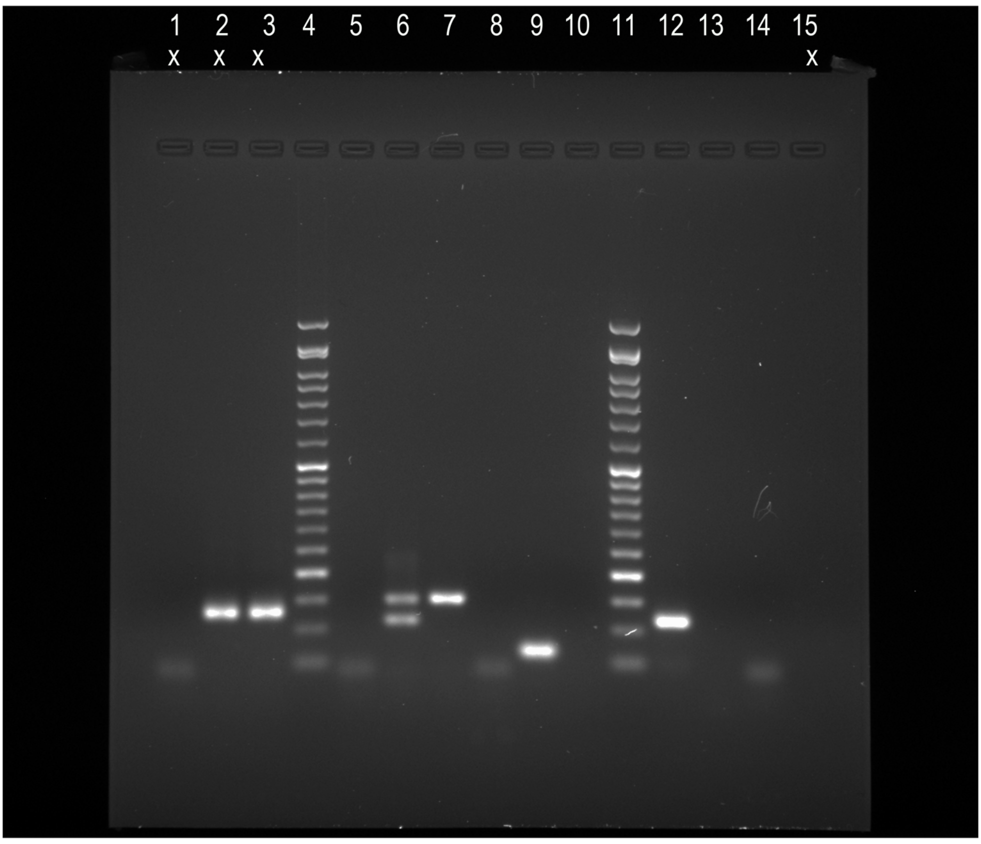

This image was partially used to generate the panel of figure 1. Agarose gel electrophoresis of amplicons generated in qPCR using primers STS154/116 (lane 5, 6, 7), TSP67 (lane 8, 9,10, overturned horizontally) and TSPY119 (lane 12,13,14) with male and female human DNA. M (lane 4,11) SharpMass™ 50 molecular weight marker - Ready-to-load DNA Ladder (Euroclone S.p.A) DNA fragments: 50,100,150,200, 250, 300, 350,400, 450,500, 600,700,800, 900, 1000, 1200 and 1500 bp. Agarose gels are 2.2% and stained with ethidium bromide as reported in Materials and Methods. Gels were acquired with the Gel Doc XR+Gel Documentation System (Bio-Rad).

S1 raw images
Figure 2

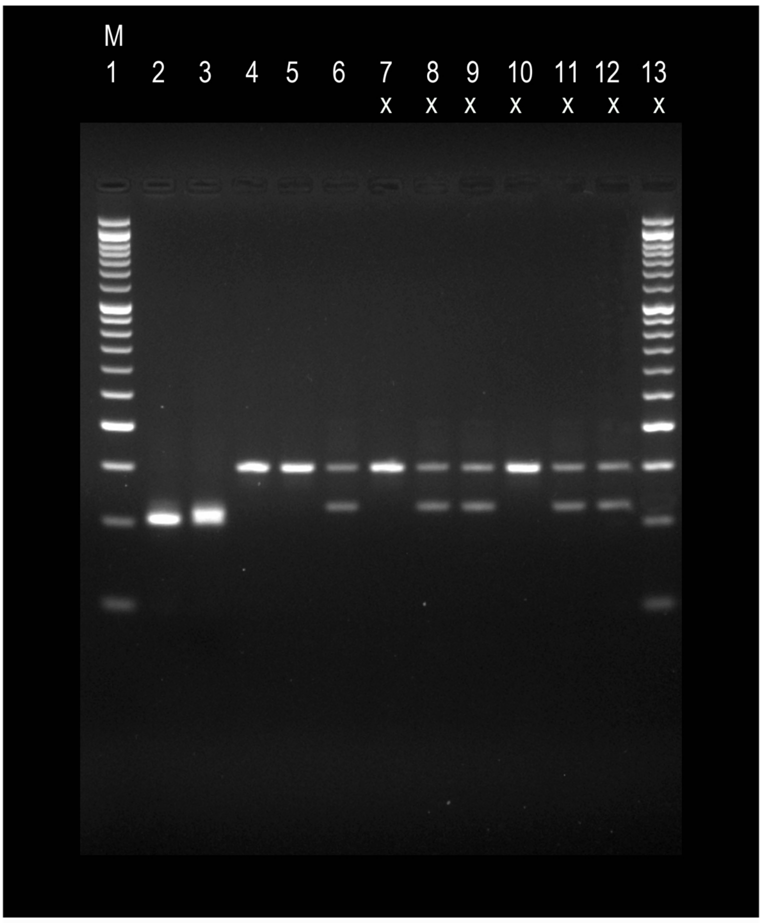

This image was partially used to generate the figure 2 panel A. Agarose gel electrophoresis of amplicons generated in qPCR using primers AMEL 106/112 (lane 2,3), STS154/116 (lane 4, 5,6) with male and female human DNA. M (lane 1) SharpMass™ 50 molecular weight marker - Ready-to-load DNA Ladder (Euroclone S.p.A) DNA fragments: 50,100,150,200, 250, 300, 350,400, 450,500, 600, 700,800, 900, 1000, 1200 and 1500 bp. Agarose gels are 2.2% and stained with ethidium bromide as reported in Materials and Methods. Gels were acquired with the Gel Doc XR+Gel Documentation System (Bio-Rad).

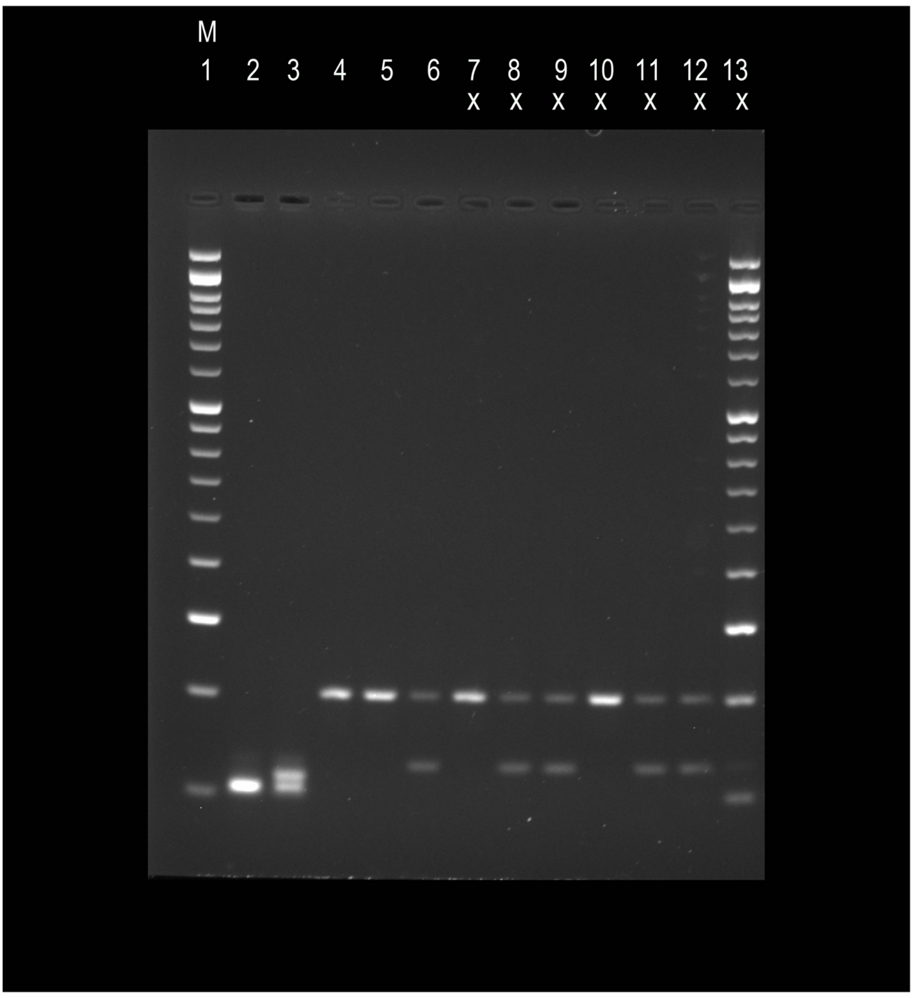

This image was partially used to generate the figure 2 panel B. Agarose gel electrophoresis of amplicons generated in qPCR using primers AMEL 106/112 (lane 2,3), STS154/116 (lane 4,5,6) with male and female human DNA. M (lane 1) SharpMass™ 50 molecular weight marker - Ready-to-load DNA Ladder (Euroclone S.p.A) DNA fragments: 50,100,150,200, 250, 300, 350,400, 450,500, 600, 700,800, 900, 1000, 1200 and 1500 bp. Agarose gels are 2.2% and stained with ethidium bromide as reported in Materials and Methods. Gels were acquired with the Gel Doc XR+Gel Documentation System (Bio-Rad).

S1 raw images
Figure 4

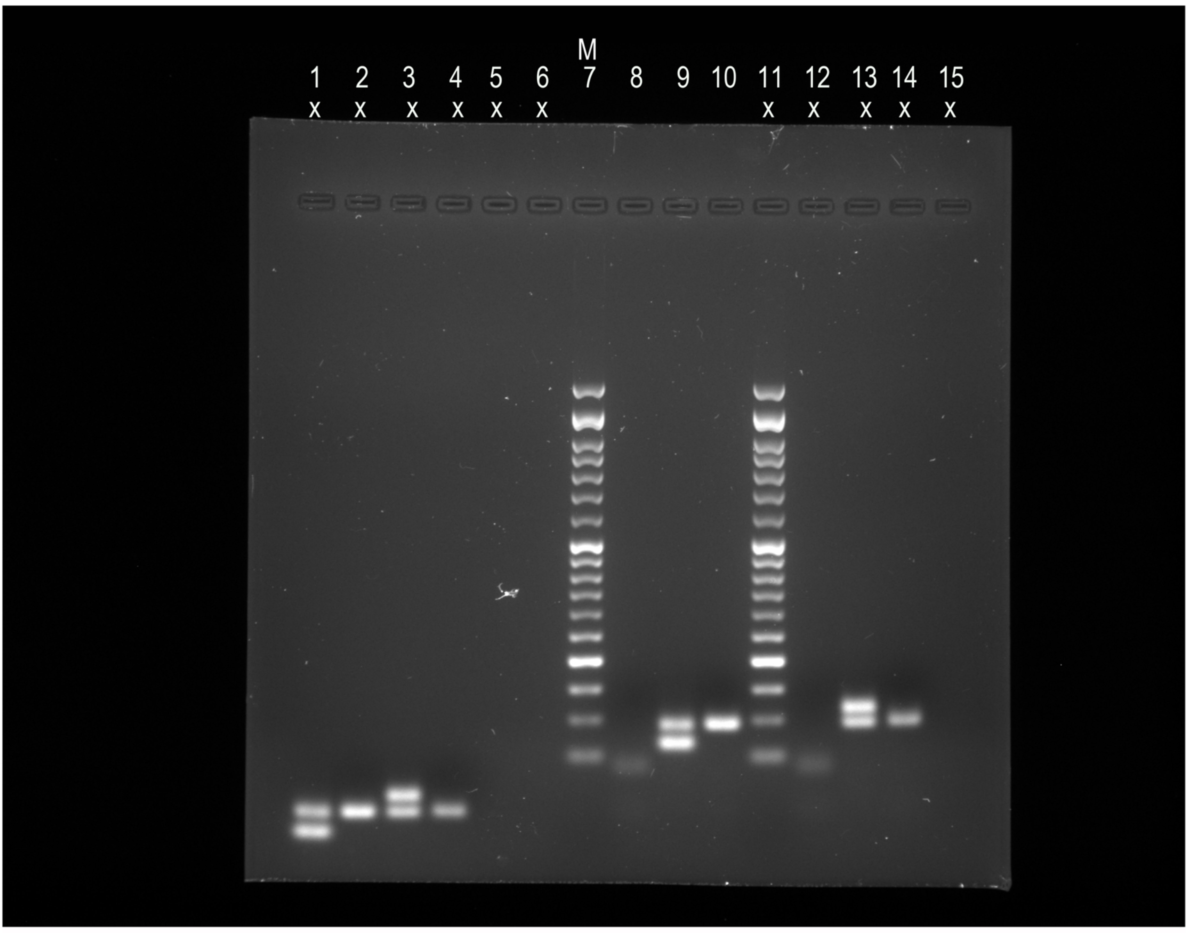

This image was partially used to generate the panel of figure 4. Agarose gel electrophoresis of amplicons generated in qPCR using primers STS95/TSPY67 (lane 8, 9, 10), with male and female human DNA. M (lane 7) SharpMass™ 50 molecular weight marker - Ready-to-load DNA Ladder (Euroclone S.p.A) DNA fragments: 50,100,150,200, 250, 300, 350,400, 450,500, 600, 700,800, 900, 1000,1200 and 1500 bp. Agarose gels are 2.2% and stained with ethidium bromide as reported in Materials and Methods. Gels were acquired with the Gel Doc XR+Gel Documentation System (Bio-Rad).

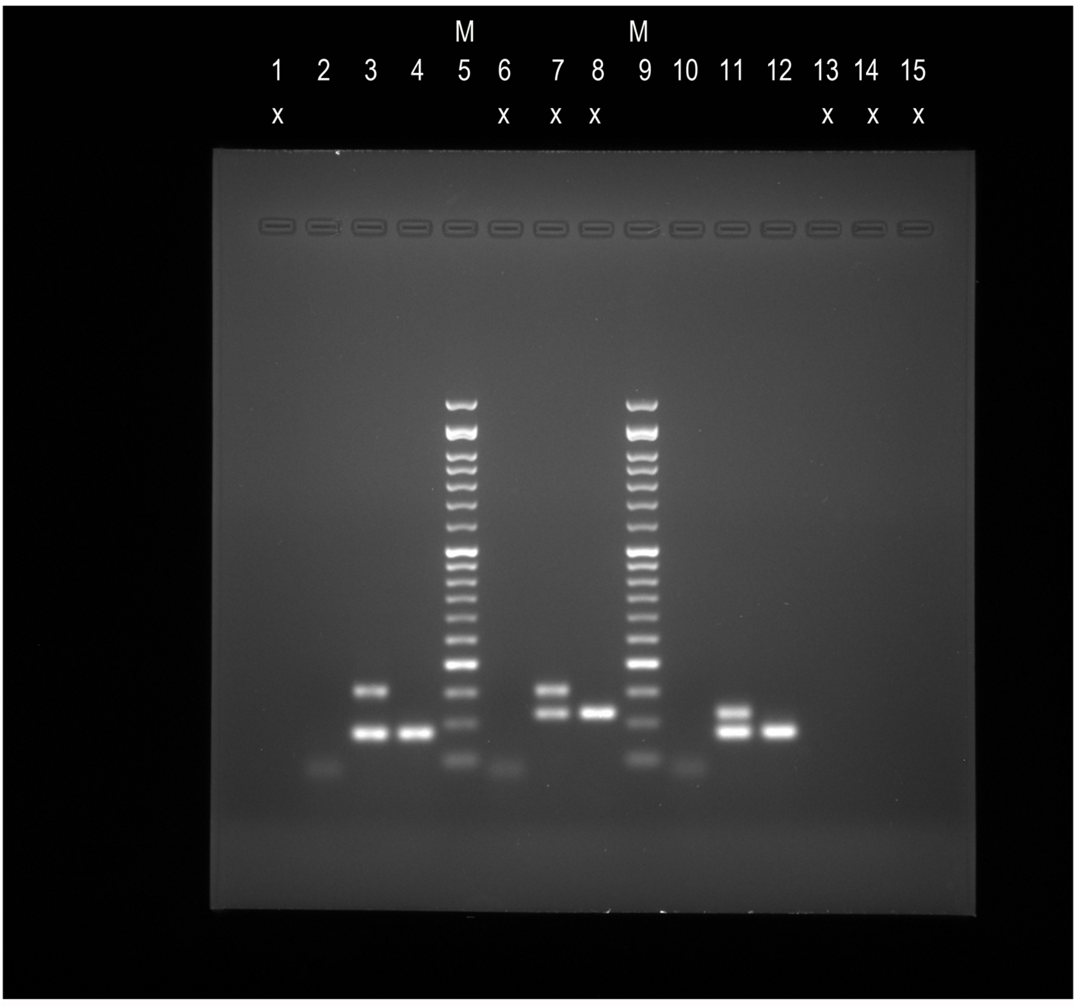

This image was partially used to generate the panel of figure 4. Agarose gel electrophoresis of amplicons generated in qPCR with modern DNA using primers STS158Y/STS89 (lane 2,3,4), STS89/TSPY119 (lane 10,11,12) with male and female human DNA. M (lane 5, 9) SharpMass™ 50 molecular weight marker - Ready-to-load DNA Ladder (Euroclone S.p.A) DNA fragments: 50,100, 150, 200, 250, 300, 350,400, 450,500, 600, 700,800, 900, 1000, 1200 and 1500 bp. Agarose gels are 2.2% and stained with ethidium bromide as reported in Materials and Methods.
Gels were acquired with the Gel Doc XR+Gel Documentation System (Bio-Rad).

S1 raw images
Figure 6

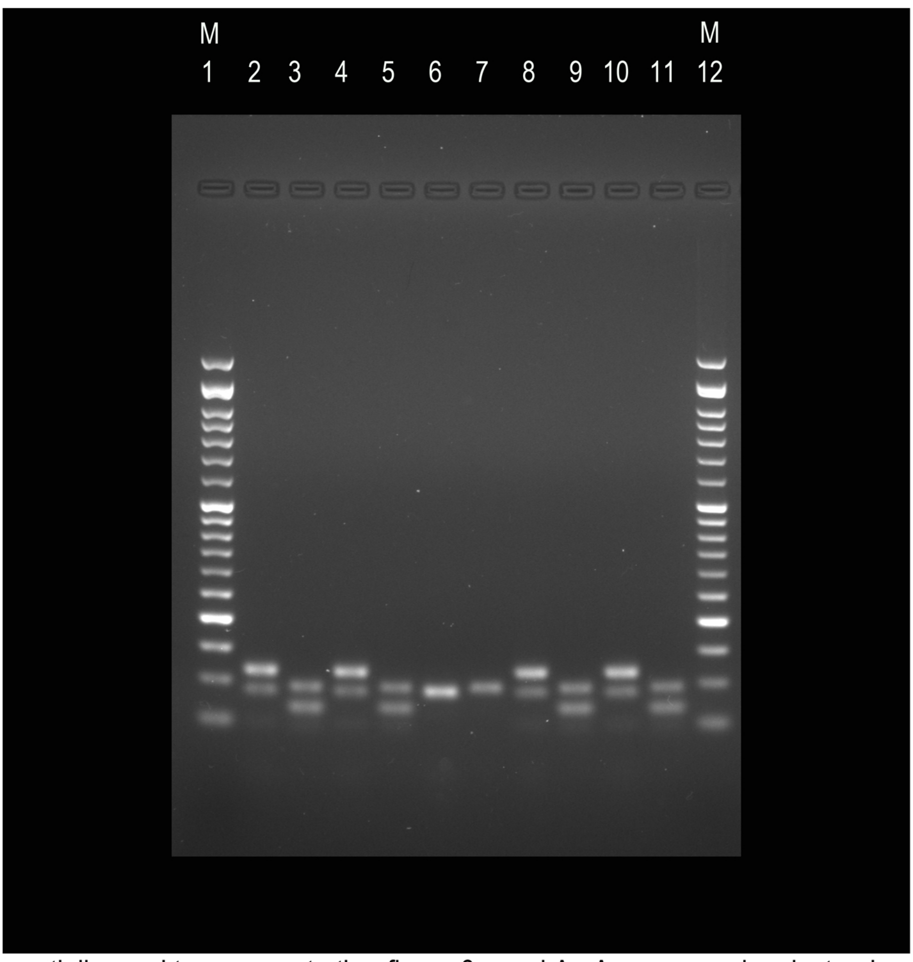

This image was partially used to generate the figure 6 panel A. Agarose gel electrophoresis of amplicons generated in qPCR using pairs of primers STS89/TSPY119, TS95/TSPY (lane 2,3,4,5,6,7,8,9,10,11) with ancient human male and female DNA. M (lane 1,12) SharpMass™ 50 molecular weight marker - Ready-to-load DNA Ladder (Euroclone S.p.A) DNA fragments: 50,100,150,200, 250, 300,350,400, 450, 500, 600, 700,800, 900, 1000, 1200 and 1500 bp. Agarose gels are 2.2% and stained with ethidium bromide as reported in Materials and Methods. Gels were acquired with the Gel Doc XR+Gel Documentation System (Bio-Rad).

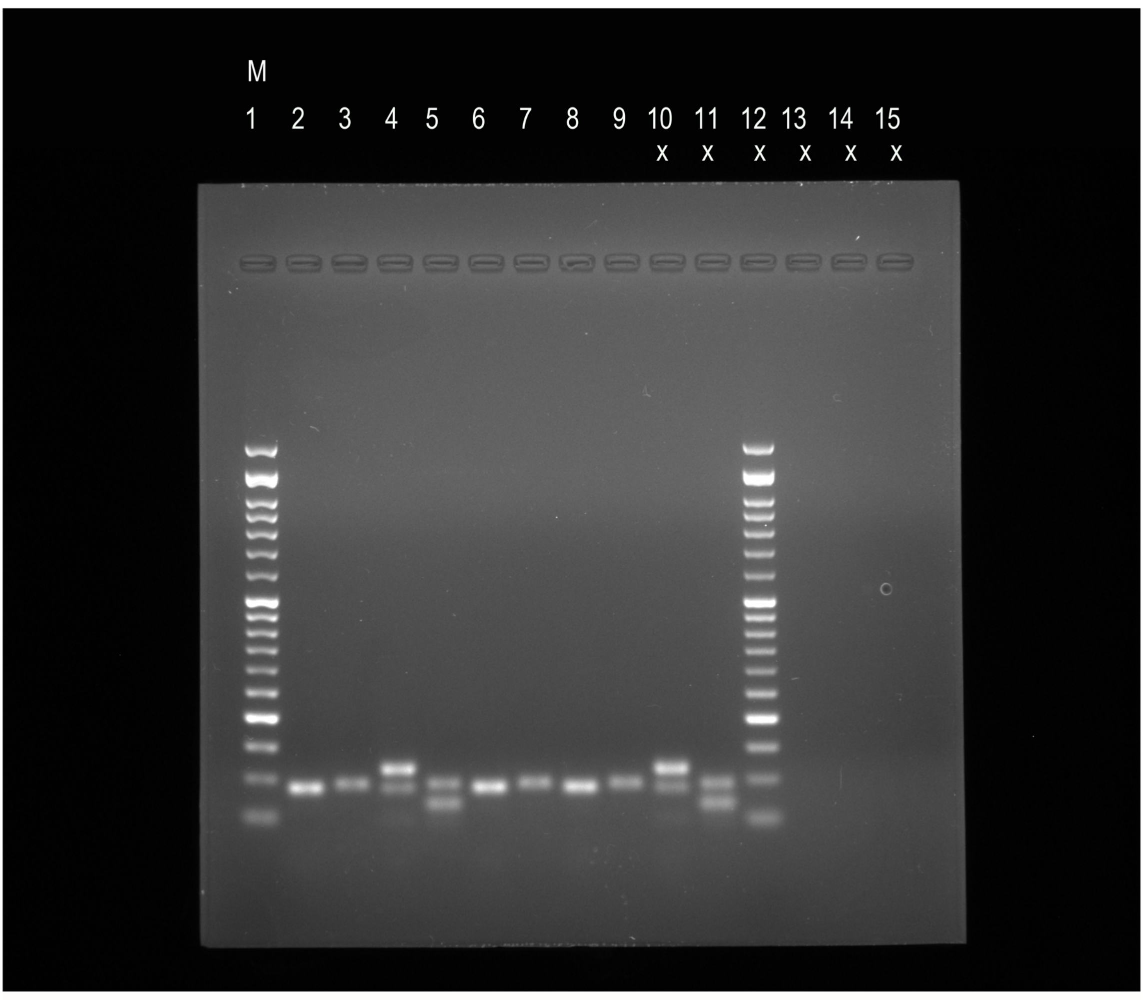

This image was partially used to generate the figure 6 panel A. Agarose gel electrophoresis of amplicons generated in qPCR using pairs primers STS89/TSPY119, STS95/TSPY67 (lane 2,3,4,5,6,7,8,9), with ancient human male and female DNA. M (lane 1) SharpMass™ 50 molecular weight marker - Ready -to-load DNA Ladder (Euroclone S.p.A) DNA fragments: 50, 100,150,200, 250, 300, 350,400, 450,500, 600, 700, 800, 900, 1000, 1200 and 1500 bp. Agarose gels are 2.2% and stained with ethidium bromide as reported in Materials and Methods. Gels were acquired with the Gel Doc XR+Gel Documentation System (Bio-Rad).

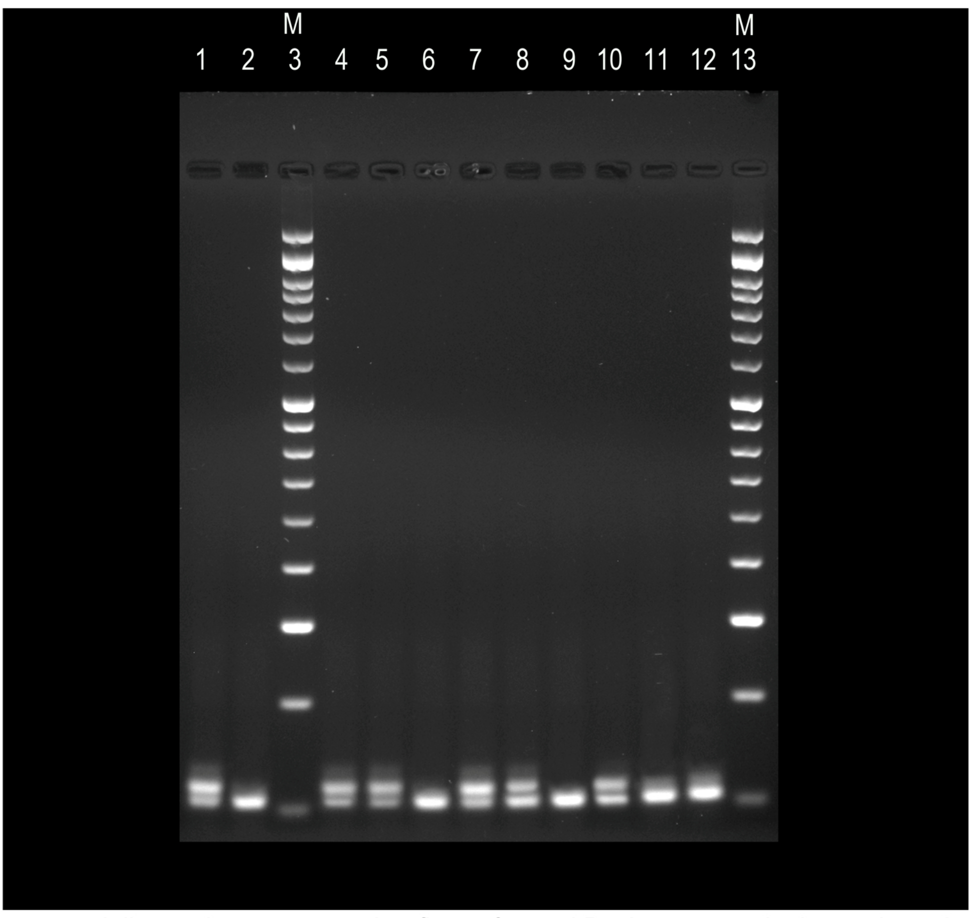

This image was partially used to generate the figure 6 panel B. Agarose gel electrophoresis of amplicons generated in qPCR using primers AMEL106/112 (lane 4,5,6,7,8,9,10,11,12), with ancient human male and female DNA. M (lane 3,13) SharpMass™ 50 molecular weight marker - Ready-to-load DNA Ladder (Euroclone S.p.A) DNA fragments: 50,100,150,200, 250, 300, 350,400, 450,500, 600, 700,800, 900, 1000, 1200 and 1500 bp. Agarose gels are 2.2% and stained with ethidium bromide as reported in Materials and Methods. Gels were acquired with the Gel Doc XR+Gel Documentation System (Bio-Rad).
